# Tubular lysosome induction couples animal starvation to healthy aging

**DOI:** 10.1038/s43587-023-00470-6

**Published:** 2023-08-14

**Authors:** Tatiana V. Villalobos, Bhaswati Ghosh, Kathryn R. DeLeo, Sanaa Alam, Cristian Ricaurte-Perez, Andrew Wang, Brennan M. Mercola, Tyler J. Butsch, Cara D. Ramos, Suman Das, Eric D. Eymard, K. Adam Bohnert, Alyssa E. Johnson

**Affiliations:** Department of Biological Sciences, Louisiana State University, Baton Rouge, LA USA

**Keywords:** Macroautophagy, Ageing, Lysosomes

## Abstract

Dietary restriction promotes longevity in several species via autophagy activation. However, changes to lysosomes underlying this effect remain unclear. Here using the nematode *Caenorhabditis elegans*, we show that the induction of autophagic tubular lysosomes (TLs), which occurs upon dietary restriction or mechanistic target of rapamycin inhibition, is a critical event linking reduced food intake to lifespan extension. We find that starvation induces TLs not only in affected individuals but also in well-fed descendants, and the presence of gut TLs in well-fed progeny is predictive of enhanced lifespan. Furthermore, we demonstrate that expression of *Drosophila* small VCP-interacting protein, a TL activator in flies, artificially induces TLs in well-fed worms and improves *C. elegans* health in old age. These findings identify TLs as a new class of lysosomes that couples starvation to healthy aging.

## Abstract (web summary)

The authors report that a change to lysosome morphology, from vesicular to tubular form, supports lifespan extension upon dietary restriction and promotes heightened autophagy and healthy aging when stimulated artificially in well-fed animals.

## Main

Lysosomes are pivotal cellular organelles that clear toxic molecular damage and recycle nutrients to maintain cellular homeostasis^1^. Their dysfunction is often catastrophic for a cell and can lead to aging and degenerative pathologies^2–4^. Given their broad implications for disease prevention and treatment, understanding how lysosomes act to support cellular and organismal health has emerged as an important topic in biomedicine. Although lysosomes have traditionally been viewed, rather simplistically, as terminal waste disposal sites, it has become apparent that lysosomes can also execute complex metabolic tasks essential to cell viability^5^. For example, lysosomes act as major signaling hubs that can sense nutrient scarcity to promote their own biogenesis^1,6–8^. Thus, lysosomes possess a remarkable versatility to sense and respond to metabolic shifts in the cell and are uniquely equipped to recalibrate cellular homeostasis on demand. But whether alternative mechanisms, in addition to increased biogenesis, can modulate lysosome activity in response to nutrient deprivation or other metabolic cues has not been fully explored.

While lysosomes are typically thought of as vesicular organelles, it has been known for some time that lysosomal membranes can form tubular projections^9,10^. Initial reports suggested that lysosome tubulation may be a rare event, aiding in non-digestive activities such as membrane recycling^11^ or in cell-specific functions such as antigen processing and presentation^12,13^. However, in more recent years, we and others have identified dynamic networks of autophagic tubular lysosomes (TLs) in various tissues and organisms^14–18^. Thus, TLs may be more broadly conserved than originally anticipated. Notably, autophagic TLs are cell type specific and, in some tissues, respond to different metabolic or developmental cues^15,16,18^. For example, TLs in the gut are robustly stimulated by starvation cues and are naturally induced during early aging to coordinate bulk age-dependent turnover of peroxisomes and potentially other autophagic cargo^17^. In *Drosophila* larval body wall muscles, TLs are constitutively present; however, increasing their density in muscles alone is sufficient to extend animal lifespan, and their disruption leads to multi-system degeneration^19^. Collectively, these studies allude to TLs as pro-health factors and indicate that, in some tissues, TLs are stimulated by nutrient deprivation. These considerations prompted us to investigate whether TLs contribute to the known health and longevity benefits of dietary restriction (DR)^20,21^.

In this Article, we demonstrate that autophagic TLs are stimulated in the *Caenorhabditis elegans* gut by multiple modes of food restriction. In this context, TL formation occurs upon mechanistic target of rapamycin (mTOR) inhibition, distinguishing it from mTOR-dependent lysosome tubulation in non-autophagic scenarios. Notably, we find that autophagic TL induction is essential for the full beneficial effects of DR and that constitutive stimulation of autophagic TLs in the gut is sufficient to promote healthy aging in wild-type worms. This TL-dependent improvement to late-age health is due, in part, to cell non-autonomous mechanisms triggered by gut TLs that elicit systemic improvements in non-gut tissues. Interestingly, the benefits brought on by TL induction can extend for several generations; while gut TLs are absent from perpetually well-fed lineages, they are induced in well-fed descendants of starved animals, conferring lifespan extension transgenerationally. Finally, we demonstrate that proteotoxic stress can also spur TL induction, and, remarkably, hyperactivation of TLs can improve cargo turnover when earlier steps in the autophagic machinery are partially compromised. On the basis of these data, we propose a model in which (1) TLs can be triggered by multiple modes of rising autophagic demands, and (2) their induction heightens degradative capacity by expanding total cellular lysosomal surface area. In principle, this morphological transformation provides more potential docking sites for cargo delivery, which becomes more crucial during nutrient deprivation or in late age when other components of the autophagic machinery begin to decline in functionality.

## Results

### Starvation and DR induce gut TLs and elevate SPIN expression

**Fig. 1:**
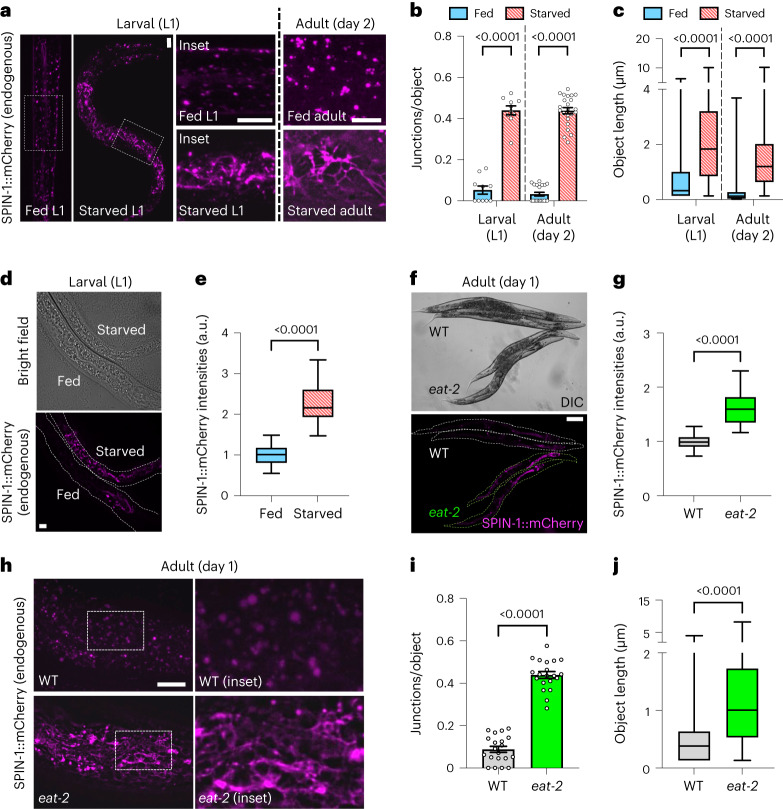
Starvation and DR induce gut TLs and elevate SPIN expression. **a**, Endogenously tagged SPIN-1::mCherry in fed and starved worms. Scale bars, 5 μm. **b**, Endogenously tagged SPIN-1::mCherry junctions/object in fed (*n* = 10 worms) and starved (*n* = 10 worms) L1 worms and in fed (*n* = 20 worms) and starved (*n* = 23 worms) day 2 adult worms. Mean ± s.e.m. Unpaired two-tailed *t*-tests with Welch’s correction. **c**, Endogenously tagged SPIN-1::mCherry object lengths in fed (*n* = 227 objects from 10 worms) and starved (*n* = 265 objects from 10 worms) L1 worms and in fed (*n* = 979 objects from 20 worms) and starved (*n* = 3,143 objects from 23 worms) day 2 adult worms. Box-and-whisker plots (minimum, 25th percentile, median, 75th percentile, maximum). Unpaired two-tailed *t*-tests with Welch’s correction. **d**, Endogenous *spin-1*::*mCherry* expression in fed and starved L1 worms. Scale bar, 5 μm. **e**, Endogenously tagged SPIN-1::mCherry fluorescence intensities in fed (*n* = 26 worms) and starved (*n* = 25 worms) L1 worms. Box-and-whisker plots (minimum, 25th percentile, median, 75th percentile, maximum). Unpaired two-tailed Student’s *t*-test. **f**, Endogenous *spin-1*::*mCherry* expression in day 1 wild-type and *eat-2* adult worms. Scale bar, 100 μm. **g**, Endogenously tagged SPIN-1::mCherry fluorescence intensities in day 1 wild-type (*n* = 31 worms) and *eat-2* (*n* = 30 worms) adult worms. Box-and-whisker plots (minimum, 25th percentile, median, 75th percentile, maximum). Unpaired two-tailed Student’s *t*-test. **h**, Endogenously tagged SPIN-1::mCherry in day 1 wild-type and *eat-2* adult worms. Scale bar, 5 μm. **i**, Endogenously tagged SPIN-1::mCherry junctions/object in day 1 wild-type (*n* = 20 worms) and *eat-2* (*n* = 20 worms) adult worms. Mean ± s.e.m. Unpaired two-tailed Student’s *t*-test. **j**, Endogenously tagged SPIN-1::mCherry object lengths in day 1 wild-type (*n* = 140 objects from 20 worms) and *eat-2* (*n* = 575 objects from 20 worms) adult worms. Box-and-whisker plots (minimum, 25th percentile, median, 75th percentile, maximum). Unpaired two-tailed *t*-test with Welch’s correction. Source data

**Extended Data Fig. 1:**
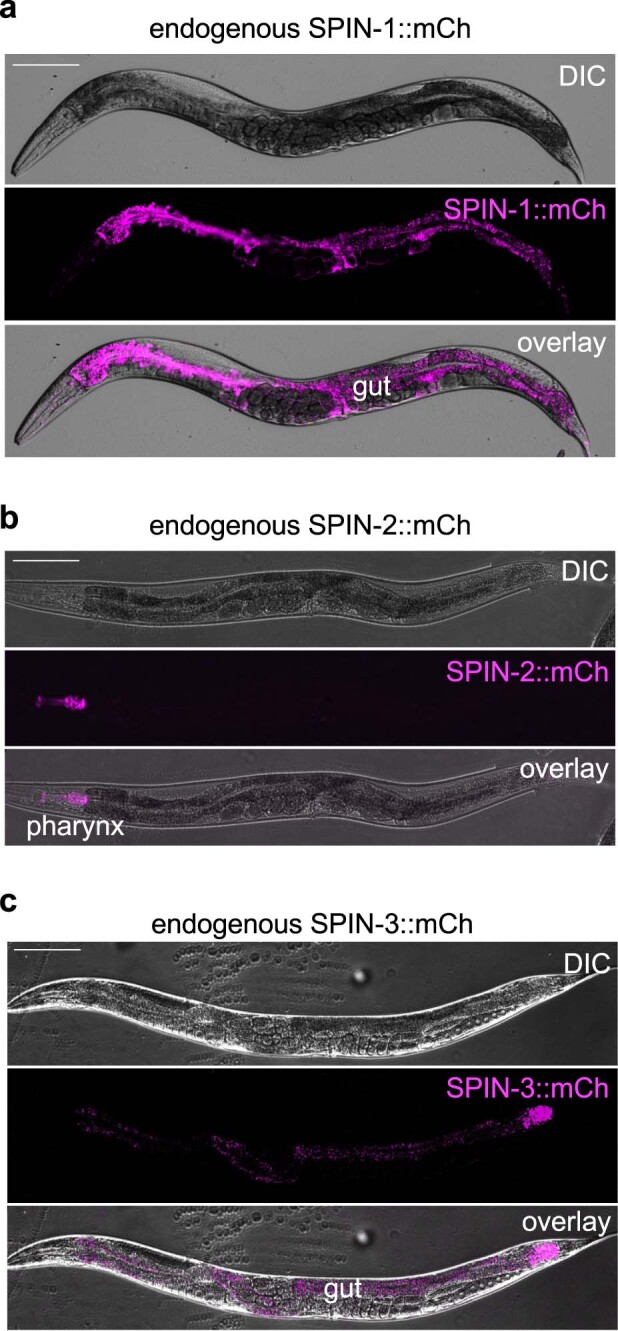
Endogenous expression patterns of *C. elegans* SPIN homologs, related to Fig. 1. (**a-c**) Endogenous expression pattern of SPIN-1::mCherry (**a**), SPIN-2::mCherry (**b**), and SPIN-3::mCherry (**c**) in day 1 adult worms (*n* = 20 worms for each condition). Bars, 100μm.

**Extended Data Fig. 2:**
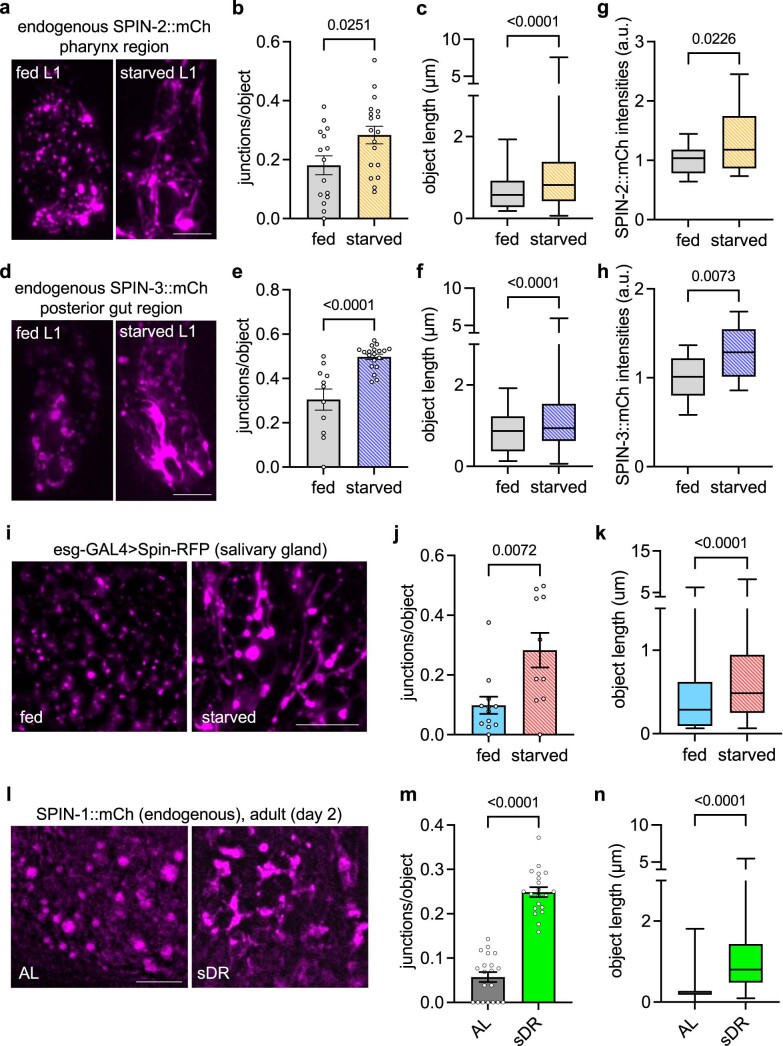
TLs are marked by various Spinster homologs in animals of different species under starvation or sDR conditions, related to Fig. 1. (**a**) Endogenously-tagged SPIN-2::mCherry in fed and starved L1 worms. Bar, 5 μm. (**b**) Endogenously-tagged SPIN-2::mCherry junctions/object in fed (*n* = 15 worms) and starved (*n* = 18 worms) L1 worms. Mean ± SEM. Unpaired two-tailed Student’s t-test. (**c**) Endogenously-tagged SPIN-2::mCherry object lengths in fed (*n* = 48 objects from 15 worms) and starved (*n* = 444 objects from 18 worms) L1 worms. Box-and-whisker plots (minimum, 25^th^ percentile, median, 75^th^ percentile, maximum). Unpaired two-tailed t-test with Welch’s correction. (**d**) Endogenously-tagged SPIN-3::mCherry in fed and starved L1 worms. Bar, 5 μm. (**e**) Endogenously-tagged SPIN-3::mCherry junctions/object in fed (*n* = 11 worms) and starved (*n* = 18 worms) L1 worms. Mean ± SEM. Unpaired two-tailed Student’s t-test. (**f**) Endogenously-tagged SPIN-3::mCherry object lengths in fed (*n* = 60 objects from 11 worms) and starved (*n* = 554 objects from 19 worms) L1 worms. Box-and-whisker plots (minimum, 25^th^ percentile, median, 75^th^ percentile, maximum). Unpaired two-tailed t-test with Welch’s correction. (**g**) Endogenously-tagged SPIN-2::mCherry fluorescence intensities in fed (*n* = 16 worms) and starved (*n* = 16 worms) L1 worms. Box-and-whisker plots (minimum, 25^th^ percentile, median, 75^th^ percentile, maximum). Unpaired two-tailed Student’s t-test. (**h**) Endogenously-tagged SPIN-3::mCherry fluorescence intensities in fed (*n* = 15 worms) and starved (*n* = 14 worms) L1 worms. Box-and-whisker plots (minimum, 25^th^ percentile, median, 75^th^ percentile, maximum). Unpaired two-tailed Student’s t-test. (**i**) Spin-RFP expressed in the salivary gland of fed and starved *Drosophila* 3rd instar larvae. Bar, 5 μm. (**j**) Spin-RFP junctions/object in fed (*n* = 12 larvae) and starved (*n* = 10 larvae) *Drosophila* 3rd instar larvae. Mean ± SEM. Unpaired two-tailed Student’s t-test. (**k**) Spin-RFP object lengths in the salivary gland of fed (*n* = 1,346 objects from 12 larvae) and starved (*n* = 2,882 objects from 10 larvae) *Drosophila* 3rd instar larvae. Box-and-whisker plots (minimum, 25^th^ percentile, median, 75^th^ percentile, maximum). Unpaired two-tailed t-test with Welch’s correction. (**l**) Endogenously-tagged SPIN-1::mCherry in day 2 adult worms fed *ad libitum* (AL) or subjected to DR on solid medium (sDR) starting from L4 larval stage. Bar, 5 µm. (**m**) Endogenously-tagged SPIN-1::mCherry junctions/object in day 2 adult worms under AL or sDR conditions (*n* = 20 worms per condition). Mean ± SEM. Unpaired two-tailed Student’s t-test. (**n**) Endogenously-tagged SPIN-1::mCherry object lengths in day 2 adult worms under AL (*n* = 69 objects from 20 worms) or sDR (*n* = 167 objects from 20 worms) conditions. Box-and-whisker plots (minimum, 25^th^ percentile, median, 75^th^ percentile, maximum). Unpaired two-tailed t-test with Welch’s correction. Source data

In *Drosophila* and *C. elegans*, TLs can be visualized in live tissues by over-expressing a fluorescently tagged lysosomal membrane protein, Spinster^14,17^. To study TLs in their most natural setting, we used clustered regularly interspaced short palindromic repeats (CRISPR) engineering to insert an mCherry tag at the endogenous C-termini of three *C. elegans* Spinster homologs: *spin-1*, *spin-2* and *spin-3*. We then examined the native expression pattern of each paralog; *spin-1* was strongly expressed throughout the intestine and in the uterus, *spin-2* was expressed in the pharynx, and *spin-3* was expressed predominantly in the posterior region of the intestine (Extended Data Fig. 1a–c). Following starvation, we found that lysosomes labeled by endogenous SPIN-1::mCherry, SPIN-2::mCherry or SPIN-3::mCherry transformed from discrete vesicles into tubular networks (Fig. 1a–c and Extended Data Fig. 2a–f), similar to our previous results using gut-specific *spin* transgenes^17^. Strikingly, endogenous SPIN-1::mCherry, SPIN-2::mCherry and SPIN-3::mCherry fluorescence intensities also increased significantly upon starvation (Fig. 1d,e and Extended Data Fig. 2g,h). Thus, *spin* expression might be under the control of starvation cues, potentially to promote TL induction. We also found that starvation induced formation of Spinster-labeled TLs in the *Drosophila* salivary gland (Extended Data Fig. 2i–k), signifying that starvation-dependent TL induction is a conserved phenomenon across multiple animal species and tissues.

We next asked if DR, like complete starvation, induces TL formation. We focused on *spin-1::mCherry*, since *spin-1* showed the strongest intestinal expression and intestinal fitness is strongly linked to the beneficial effects of DR^20^. Using *eat-2* mutants, which provide a genetic model for DR due to decreased pharyngeal pumping^21^, we found that TLs and SPIN-1::mCherry intensity indeed increased when adults were calorically restricted (Fig. 1f–j). Moreover, a similar shift from vesicular lysosome morphology to a TL network occurred when wild-type *C. elegans* were subjected to DR by an approach that restricts the amount of food given to animals on solid medium (solid dietary restriction, sDR)^22^ (Extended Data Fig. 2l–n). These data collectively indicate that gut TLs are robustly stimulated by multiple methods of food restriction.

### Gut TLs are autophagic and stimulated by mTOR inhibition

**Fig. 2:**
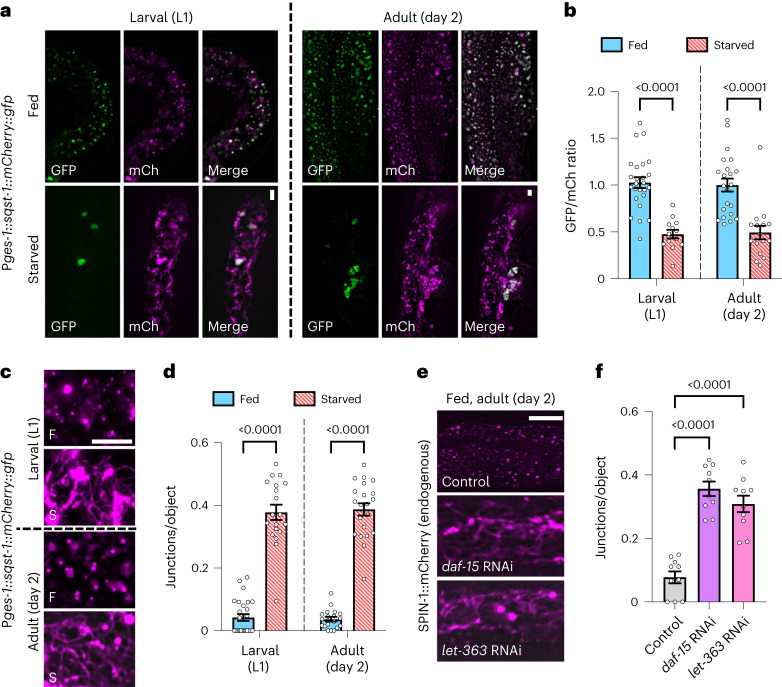
Gut TLs are autophagic and stimulated by mTOR inhibition. **a**, Gut-expressed SQST-1::mCherry::GFP in fed and starved L1 and day 2 adult worms. Scale bars, 5 μm. **b**, GFP/mCherry ratio of gut-expressed SQST-1::mCherry::GFP in fed (*n* = 26 worms) and starved (*n* = 13 worms) L1 worms and in fed (*n* = 22 worms) and starved (*n* = 16 worms) day 2 adult worms. Mean ± s.e.m. Unpaired two-tailed Student’s *t*-tests. **c**, Gut-expressed SQST-1::mCherry::GFP red (mCherry) fluorescence in fed (F) and starved (S) L1 and day 2 adult worms. Scale bar, 5 μm. **d**, Junctions/object for mCherry-labeled objects in fed (*n* = 27 worms) and starved (*n* = 19 worms) *sqst-1::mCherry::gfp* L1 worms and in fed (*n* = 19 worms) and starved (*n* = 21 worms) *sqst-1::mCherry::gfp* day 2 adult worms. Mean ± s.e.m. Unpaired two-tailed Student’s *t*-tests. **e**, Endogenously tagged SPIN-1::mCherry in day 2 adult worms fed control, *daf-15* or *let-363* RNAi. Scale bar, 5 μm. **f**, Endogenously tagged SPIN-1::mCherry junctions/object in day 2 adult worms fed control, *daf-15* or *let-363* RNAi (*n* = 10 worms per condition). Mean ± s.e.m. One-way ANOVA with Dunnett’s multiple comparisons. Source data

**Extended Data Fig. 3:**
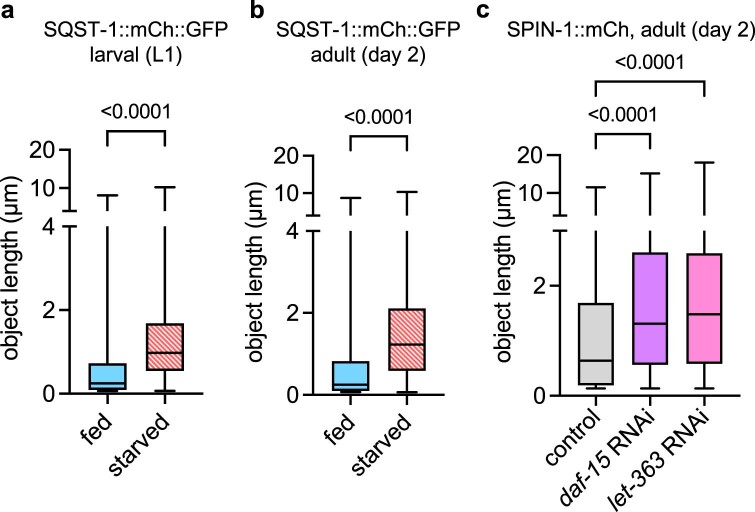
Gut TLs are autophagic and stimulated by mTOR inhibition, related to Fig. 2. (**a**) Object lengths for mCherry-labeled objects in fed (*n* = 278 objects from 27 worms) and starved (*n* = 667 objects from 19 worms) *sqst-1::mCherry::gfp* L1 worms. Box-and-whisker plots (minimum, 25^th^ percentile, median, 75^th^ percentile, maximum). Unpaired two-tailed t-test with Welch’s correction. (**b**) Object lengths for mCherry-labeled objects in fed (*n* = 186 objects from 21 worms) and starved (*n* = 270 objects from 19 worms) *sqst-1::mCherry::gfp* day 2 adult worms. Box-and-whisker plots (minimum, 25^th^ percentile, median, 75^th^ percentile, maximum). Unpaired two-tailed t-test with Welch’s correction. (**c**) Endogenously-tagged SPIN-1::mCherry object lengths in day 2 adult worms treated with control (*n* = 435 objects from 10 worms), *daf-15* (*n* = 920 objects from 10 worms), or *let-363* (*n* = 627 objects from 10 worms) RNAi. Box-and-whisker plots (minimum, 25^th^ percentile, median, 75^th^ percentile, maximum). One-way ANOVA with Dunnett’s multiple comparisons. Source data

Nutrient deprivation is a major autophagic stimulant^23–25^. We and others have previously reported that TLs can be autophagic^14–17^, though TLs in separate contexts may be nondegradative^26^. To clarify if TLs induced by starvation are in fact major autophagic sites, we expressed SQST-1/p62, an autophagy receptor^27^, with a tandem mCherry::green fluorescent protein (GFP) epitope, in which only the GFP fluorescence is sensitive to lysosomal pH^28^. Consistent with our prior observations of starvation-induced degradation of peroxisomes at TLs^17^, we found that green fluorescence of the SQST-1::mCherry::GFP reporter decreased relative to red after starvation (Fig. 2a,b), indicative of GFP signal quenching inside the acidic lysosomal lumen^28^, and the remaining red fluorescence redistributed into tubules (Fig. 2c,d and Extended Data Fig. 3a,b). These data fit with starvation-induced TLs being generally autophagic.

Because starvation-based autophagy induction relies on inhibition of mTOR signaling^29–31^, we tested whether TL induction was triggered upon mTOR inhibition. Indeed, *let-363* (mTOR) and *daf-15* (RPTOR) gene knockdowns, either of which alone can extend longevity^32,33^, induced TLs in the gut of well-fed animals (Fig. 2e,f and Extended Data Fig. 3c). Thus, starvation-induced TLs target autophagic cargo and are stimulated by mTOR inhibition. This is a notable distinction from the lysosome tubules that have been previously described to function during autophagic lysosome reformation (ALR), whereby proto-lysosome tubules emanate from autolysosomes as a mechanism to replenish the pool of functional lysosomes after autophagy terminates^11^. The proto-lysosome tubules generated via ALR are devoid of autophagic cargo, are not acidic and require mTOR re-activation for their induction^11^. In contrast, the intestinal TLs we observe are triggered during autophagy to internalize autophagic cargo (Fig. 2a–d and Extended Data Fig. 3a,b), are acidic^17^ and are stimulated by mTOR inhibition (Fig. 2e,f and Extended Data Fig. 3c), rather than by mTOR activation. Thus, starvation-induced TLs are distinct from ALR and are associated with active autophagy.

### TL induction is independent of autophagosome assembly

**Fig. 3:**
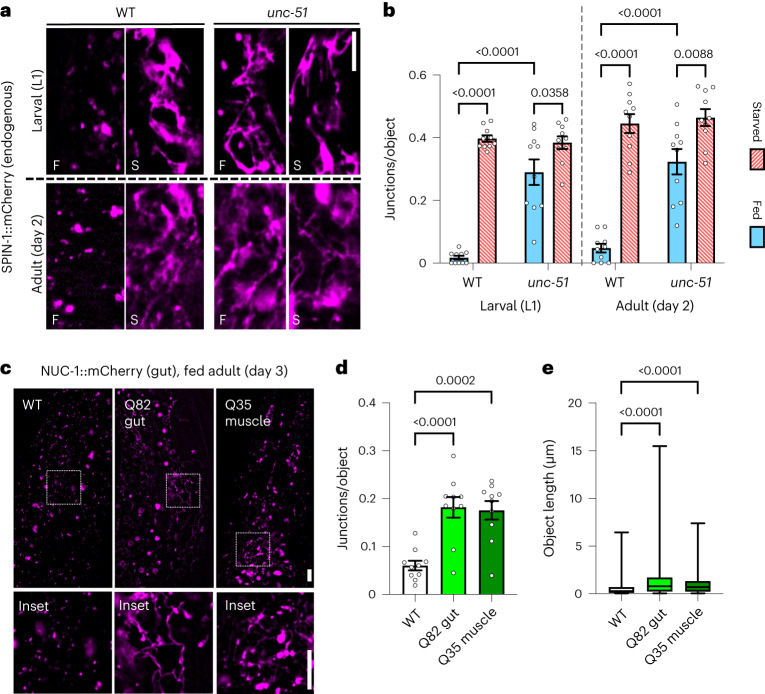
TL induction is independent of autophagosome assembly. **a**, Endogenously tagged SPIN-1::mCherry in fed (F) and starved (S) wild-type and *unc-51* worms at L1 larval stage and at day 2 of adulthood. Scale bar, 5 μm. **b**, Endogenously tagged SPIN-1::mCherry junctions/object in fed and starved wild-type and *unc-51* worms at L1 larval stage and at day 2 of adulthood (*n* = 10 worms per condition). Mean ± s.e.m. Two-way ANOVA with Tukey’s multiple comparisons. **c**, Gut-expressed NUC-1::mCherry in day 3 wild-type adult worms, in day 3 adult worms over-expressing gut Q82::YFP, and in day 3 adult worms over-expressing muscle Q35::YFP. Scale bar, 5 μm. **d**, Gut-expressed NUC-1::mCherry junctions/object in day 3 wild-type adult worms (*n* = 10 worms), in day 3 adult worms over-expressing gut Q82::YFP (*n* = 10 worms), and in day 3 adult worms over-expressing muscle Q35::YFP (*n* = 10 worms). Mean ± s.e.m. One-way ANOVA with Dunnett’s multiple comparisons. **e**, Gut-expressed NUC-1::mCherry object lengths in day 3 wild-type adult worms (*n* = 1,367 objects from 10 worms), in day 3 adult worms over-expressing gut Q82::YFP (*n* = 1,264 objects from 10 worms) and in day 3 adult worms over-expressing muscle Q35::YFP (*n* = 1,119 objects from 10 worms). Box-and-whisker plots (minimum, 25th percentile, median, 75th percentile, maximum). One-way ANOVA with Dunnett’s multiple comparisons. Source data

**Extended Data Fig. 4:**
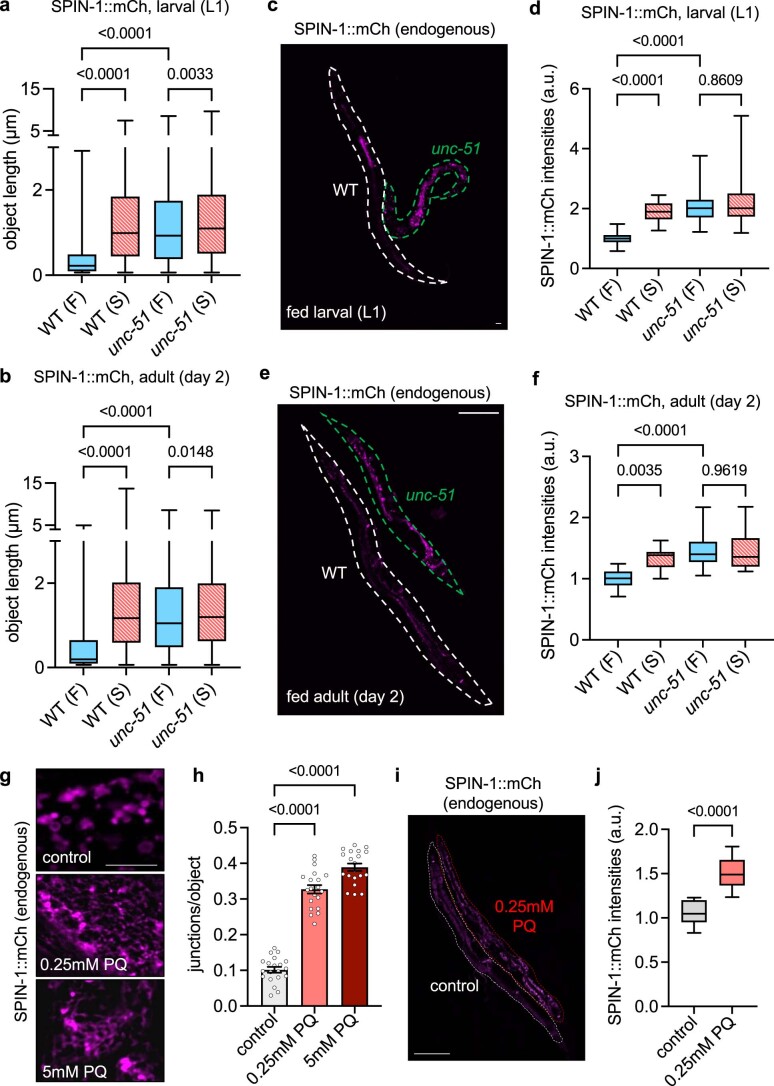
Gut TLs are stimulated upon inhibition of autophagosome assembly or oxidative stress, related to Fig. 3. (**a**) Endogenously-tagged SPIN-1::mCherry object lengths in fed (*n* = 419 objects from 10 worms) and starved (*n* = 1,321 objects from 10 worms) wild-type and fed (*n* = 1,402 objects from 10 worms) and starved (*n* = 1,696 objects from 10 worms) *unc-51* worms at L1 larval stage. F = Fed, S = Starved. Box-and-whisker plots (minimum, 25^th^ percentile, median, 75^th^ percentile, maximum). Two-way ANOVA with Tukey’s multiple comparisons. (**b**) Endogenously-tagged SPIN-1::mCherry object lengths in fed (*n* = 487 objects from 10 worms) and starved (*n* = 1,446 objects from 10 worms) wild-type and fed (*n* = 1,767 objects from 10 worms) and starved (*n* = 2,185 objects from 10 worms) *unc-51* worms at day 2 of adulthood. F = Fed, S = Starved. Box-and-whisker plots (minimum, 25^th^ percentile, median, 75^th^ percentile, maximum). Two-way ANOVA with Tukey’s multiple comparisons. (**c**) Endogenous *spin-1::mCherry* expression in fed wild-type and *unc-51* L1 worms. Bar, 5 µm. (**d**) Endogenously-tagged SPIN-1::mCherry fluorescence intensities in fed (*n* = 34 worms) and starved (*n* = 32 worms) wild-type L1 worms and in fed (*n* = 35 worms) and starved (*n* = 35 worms) *unc-51* L1 worms. F = Fed, S = Starved. Box-and-whisker plots (minimum, 25^th^ percentile, median, 75^th^ percentile, maximum). Two-way ANOVA with Tukey’s multiple comparisons. (**e**) Endogenous *spin-1::mCherry* expression in fed wild-type and *unc-51* day 2 adult worms. Bar, 100 µm. (**f**) Endogenously-tagged SPIN-1::mCherry fluorescence intensities in fed and starved wild-type and fed and starved *unc-51* day 2 adult worms (*n* = 15 worms per condition). F = Fed, S = Starved. Box-and-whisker plots (minimum, 25^th^ percentile, median, 75^th^ percentile, maximum). Two-way ANOVA with Tukey’s multiple comparisons. (**g**) Endogenously-tagged SPIN-1::mCherry in worms treated with control (0 mM), low (0.25 mM), or high (5 mM) doses of Paraquat (PQ). Bar, 5 µm. (**h**) Endogenously-tagged SPIN-1::mCherry junctions/object (*n* = 20 worms per condition). Mean ± SEM. One-way ANOVA with Dunnett’s multiple comparisons. (**i**) Endogenous *spin-1::mCherry* expression in worms treated with control or 0.25 mM PQ. Bar, 100 µm. (**j**) Endogenously-tagged SPIN-1::mCherry fluorescence intensities after PQ treatment (*n* = 20 worms per condition). Box-and-whisker plots (minimum, 25^th^ percentile, median, 75^th^ percentile, maximum). Unpaired two-tailed Student’s t-test. Source data

Currently, there are conflicting data in the literature regarding whether TL formation requires autophagosome fusion with lysosomes^14,16^. Using the endogenous SPIN-1::mCherry marker, we assessed if TLs still formed upon starvation in *unc-51*/*atg-1* mutants, which lack function of the most upstream factor involved in autophagosome formation^34,35^. Surprisingly, not only did we observe TLs in starved *unc-51* mutants, but we also observed TL induction in well-fed *unc-51* animals (Fig. 3a,b and Extended Data Fig. 4a,b). Consistent with constitutive TL induction, well-fed *unc-51* larval and adult animals showed higher endogenous SPIN-1::mCherry fluorescence intensities compared to wild-type controls (Extended Data Fig. 4c–f). These data indicate that starvation-induced TL formation does not require autophagosome membrane fusion.

Our observation that dysfunction in autophagosome formation can itself drive TL formation suggests that a stimulus from uncleared or accumulated cargo may be sufficient to trigger this morphological transformation in lysosomes independent of cargo delivery. To test this directly, we experimentally induced protein aggregates by over-expressing polyQ proteins in the gut of young well-fed animals and visualized lysosomes using the lysosomal nuclease NUC-1::mCherry marker, which also robustly marks TLs when they form^15^. Indeed, over-expression of Q82::YFP in the gut was sufficient to induce gut TLs (Fig. 3c–e), indicating that increased proteotoxic stress can serve as a TL stimulant. Consistent with this interpretation, treatment with Paraquat, an oxidizing agent that can induce in vivo protein aggregation^36,37^, likewise resulted in TL induction (Extended Data Fig. 4g–j). Remarkably, when we over-expressed Q35::YFP in a separate tissue (muscle), we also observed TL induction in the guts of young well-fed animals (Fig. 3c–e). Thus, TLs can be stimulated by both cell autonomous and non-autonomous proteotoxic cues.

### Gut TLs are induced transgenerationally

**Fig. 4:**
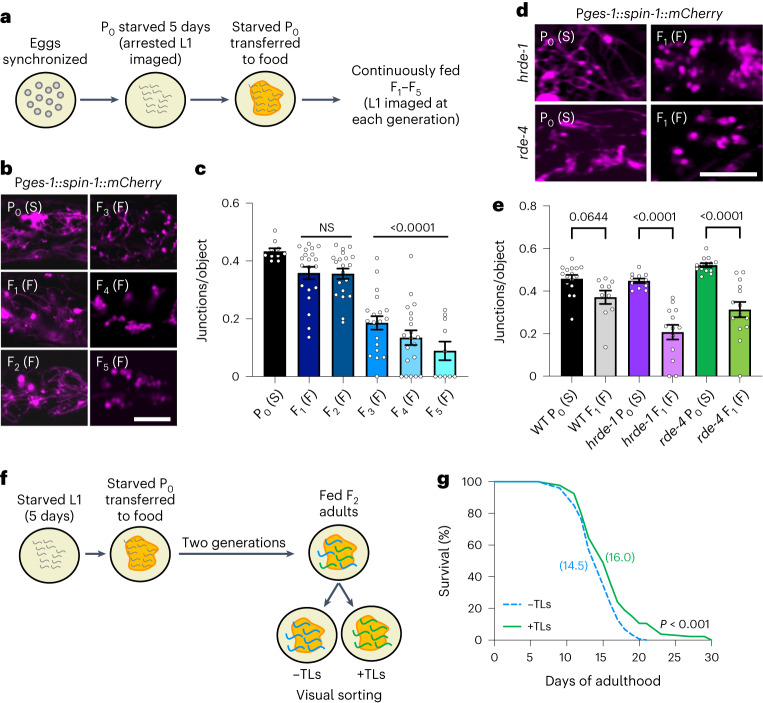
Gut TLs are induced transgenerationally. **a**, Synchronized eggs were seeded onto NGM agar with no food source. After 5 days, starved L1-arrested worms were transferred to food, and worm populations were continuously fed and imaged for all subsequent generations. **b**, Gut-expressed SPIN-1::mCherry in starved (S) L1 worms and in subsequent fed (F) generations. P_0_ and F_1_–F_5_ indicate generations. Scale bar, 5 μm. **c**, Gut-expressed SPIN-1::mCherry junctions/object in starved (S) P_0_ (*n* = 10 worms) and fed (F) F_1_–F_5_ L1 worms (*n* = 10 worms for fed F_5_, and *n* = 20 worms for each other fed generation). P_0_ and F_1_–F_5_ indicate generations. Mean ± s.e.m. One-way ANOVA with Dunnett’s multiple comparisons (*P* values on graph indicate significance compared to P_0_ (S) condition). NS, not significant. **d**, Gut-expressed SPIN-1::mCherry in starved (S) P_0_ and fed (F) F_1_ *hrde-1* and *rde-4* mutants defective in siRNA production and transmission, respectively. P_0_ and F_1_ indicate generations. Scale bar, 5 μm. **e**, Gut-expressed SPIN-1::mCherry junctions/object in starved P_0_ wild-type (*n* = 16 worms), fed F_1_ wild-type (*n* = 10 worms), starved P_0_ *hrde-1* (*n* = 11 worms), fed F_1_ *hrde-1* (*n* = 13 worms), starved P_0_ *rde-4* (*n* = 14 worms) and fed F_1_ *rde-4* (*n* = 14 worms) L1 worms. P_0_ and F_1_ indicate generations. F, fed; S, starved. Mean ± s.e.m. One-way ANOVA with Šídák’s multiple comparisons. **f**, Synchronized eggs were seeded onto NGM agar with no food source. After 5 days, starved L1-arrested worms were transferred to food, and worm populations were continuously fed for 2 generations before visual sorting. **g**, Lifespan of progeny from starved grandparents with (+) or without (−) TLs. Mean lifespans are indicated on the graph. A log-rank test was used to determine statistical significance (*P* value on graph). For statistics, see Extended Data Fig. 5c,d. Source data

A striking phenomenon of nutrient deprivation is that the beneficial physiological effects can persist for multiple generations; descendants of starved parents exhibit similar lifespan extension, despite never encountering starvation themselves^38,39^. This prompted us to explore whether TLs also persist in subsequent generations of starved parents. Synchronized embryos were seeded onto plates with no food, inducing an L1 larval arrest. After 5 days of starvation, arrested L1 larvae were transferred to food, allowing their development to resume to adulthood (Fig. 4a). The offspring of these starved parents were then imaged. Remarkably, F_1_ progeny displayed similar levels of TLs compared to their parents despite being well fed (Fig. 4b,c). This effect persisted for up to four generations, but the penetrance diminished significantly by the F_3_ generation (Fig. 4b,c).

**Extended Data Fig. 5:**
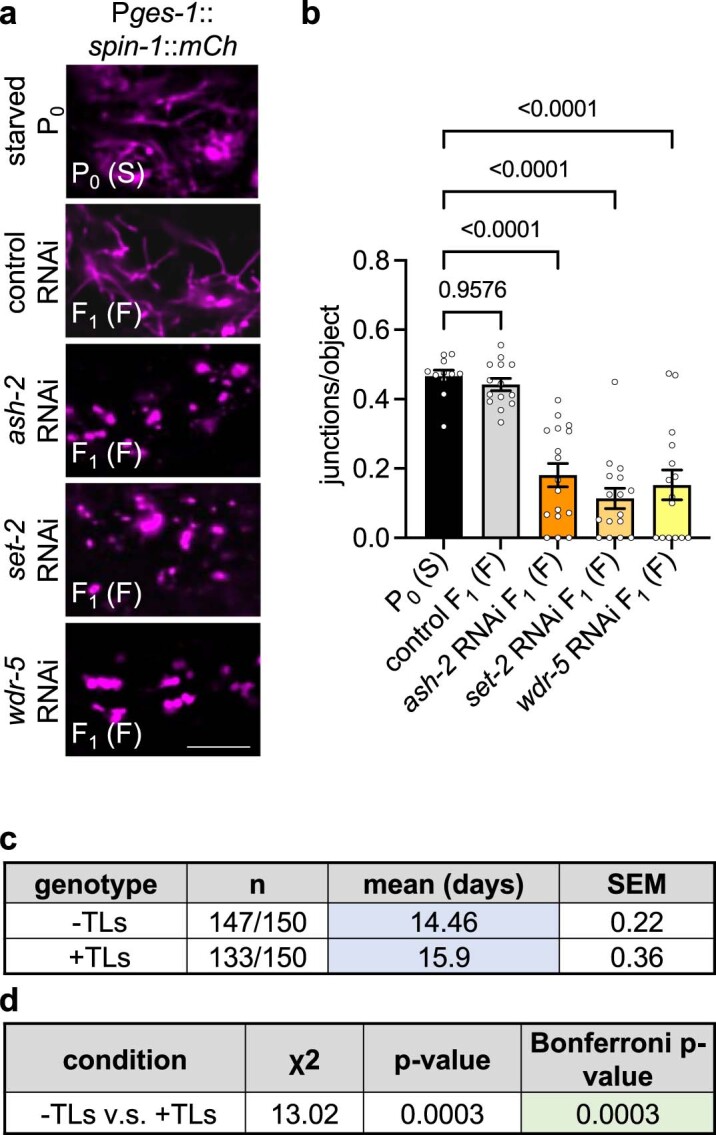
Transgenerational induction of TLs requires H3K4me3, related to Fig. 4. (**a**) Gut-expressed SPIN-1::mCherry in starved P_0_ L1 worms and in fed F_1_ L1 worms following control, *ash-2*, *set-2*, or *wdr-5* RNAi. P_0_ and F_1_ indicate generations. F = Fed, S = Starved. Bar, 5 µm. (**b**) Gut-expressed SPIN-1::mCherry junctions/object in starved P_0_ (*n* = 11 worms), fed control F_1_ (*n* = 14 worms), fed *ash-2* RNAi F_1_ (*n* = 17 worms), fed *set-2* RNAi F_1_ (*n* = 16 worms), and fed *wdr-5* RNAi F_1_ (*n* = 15 worms) L1 worms. P_0_ and F_1_ indicate generations. F = Fed, S = Starved. Mean ± SEM. One-way ANOVA with Dunnett’s multiple comparisons. (**c**) Descriptive statistics for lifespans in Fig. 4g. (**d**) Log-rank test results for lifespan comparisons in Fig. 4g. Source data

One mechanism by which starvation elicits transgenerational effects in *C. elegans* is by inducing expression of silencing RNAs (siRNAs), which persist throughout life and transmit epigenetic information to the next generation^38^. We tested whether transgenerational induction of TLs after starvation is also dependent on siRNAs by examining TLs in the gut of *rde-4* and *hrde-1* mutant worms, which are defective in siRNA production and transmission, respectively^40,41^. In both mutants, we observed that TLs were no longer robustly transmitted even in the immediate offspring of starved animals (Fig. 4d,e), indicating that this transgenerational effect indeed requires siRNAs. Transgenerational epigenetic inheritance also commonly involves changes to histone modifications. In particular, the histone H3 lysine 4 trimethylation (H3K4me3) complex regulates transgenerational effects on longevity in *C. elegans*^42^ and is required to transfer pro-health capabilities, including stress resistance, from one generation to the next in *C. elegans* and mice^43–45^. We found that knockdown of three *C. elegans* H3K4me3 complex genes, *ash-2*, *wdr-5* and *set-2*, also impaired transgenerational TL induction following starvation (Extended Data Fig. 5a,b). These findings suggest that histone methylation may also play an important role in facilitating the transgenerational effects of starvation, consistent with recent tracking of heritable methylation across generations following starvation^46^.

Because the penetrance of transgenerational TL induction decreased gradually with time (Fig. 4b,c), there was variation among descendants within the same generation; some animals displayed robust TLs while others did not. Variation in transgenerational TL induction with each generation could be reflective of differences in epigenetic transmission, cycling or reversion in each individual animal^47–49^. Similarly, other forms of epigenetic inheritance in *C. elegans* have also been shown to diminish gradually with each generation^50^. Therefore, we wondered whether transgenerational induction of TLs could provide a predictive marker to distinguish longer-lived individuals within a single generation that came from the same population of starved ancestors. To test this, we sorted F_2_ descendants derived from starved grandparents based on the visual presence or absence of robust TLs in the gut (Fig. 4f). We then compared the lifespan of these sibling populations. Remarkably, we found that F_2_ worms that retained TLs lived significantly longer (*P* < 0.001) than their sibling cohorts without TLs (Fig. 4g and Extended Data Fig. 5c,d). Taken together, our data demonstrate that starvation-induced TLs persist for multiple generations dependent on siRNAs and H3K4me3 modifications and may provide a predictive cellular marker for longer-lived individuals.

### TL induction supports DR-dependent longevity

**Fig. 5:**
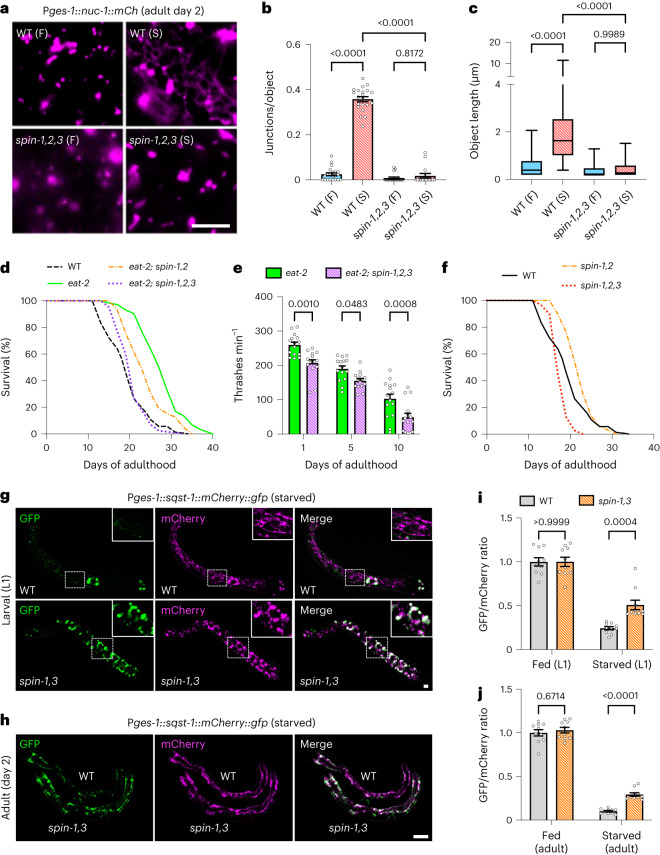
TL induction supports DR-dependent longevity. **a**, Gut-expressed NUC-1::mCherry in fed (F) and starved (S) wild-type (WT) and *spin-1,2,3* day 2 adult worms. Scale bar, 5 μm. **b**, Gut-expressed NUC-1::mCherry junctions/object in fed (*n* = 21 worms) and starved (*n* = 21 worms) wild-type day 2 adult worms and in fed (*n* = 18 worms) and starved (*n* = 15 worms) *spin-1,2,3* day 2 adult worms. F, fed; S, starved. Mean ± s.e.m. Two-way ANOVA with Tukey’s multiple comparisons. **c**, Gut-expressed NUC-1::mCherry object lengths in fed (*n* = 555 objects from 21 worms) and starved (*n* = 1,026 objects from 21 worms) wild-type day 2 adult worms and in fed (*n* = 155 objects from 18 worms) and starved (*n* = 162 objects from 15 worms) *spin-1,2,3* day 2 adult worms. Box-and-whisker plots (minimum, 25th percentile, median, 75th percentile, maximum). Two-way ANOVA with Tukey’s multiple comparisons. **d**, Lifespan of the indicated genotypes. A log-rank test was used to determine statistical significance. For statistics, see Extended Data Fig. 7b,c. **e**, Thrashing rate of *eat-2* and *eat-2*; *spin-1,2,3* mutants (*n* = 15 worms per condition). Mean ± s.e.m. Two-way ANOVA with Šídák’s multiple comparisons. **f**, Lifespan of the indicated genotypes. A log-rank test was used to determine statistical significance. For statistics, see Extended Data Fig. 7b,c. **g**, Gut-expressed SQST-1::mCherry::GFP in starved wild-type and *spin-1,2,3* L1 worms. Scale bar, 5 μm. Enlarged insets are shown to demonstrate lysosome morphology. **h**, Gut-expressed SQST-1::mCherry::GFP in starved wild-type and *spin-1,3* day 2 adult worms. Scale bar, 100 μm. **i**,**j**, GFP/mCherry ratio of gut-expressed SQST-1::mCherry::GFP in fed and starved wild-type and *spin-1,2,3* L1 worms (**i**) and in fed and starved wild-type and *spin-1,3* day 2 adult worms (**j**) (*n* = 10 worms per condition). Mean ± s.e.m. Two-way ANOVA with Šídák’s multiple comparisons. Source data

**Extended Data Fig. 6:**
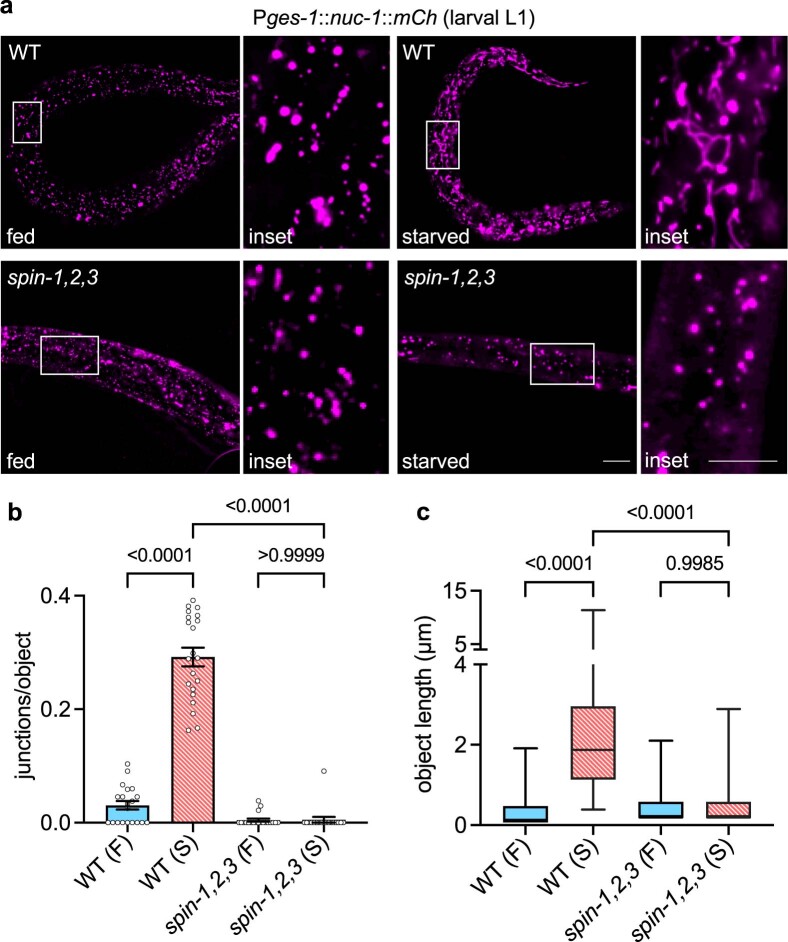
Gut TL induction is dependent on *C. elegans* SPIN proteins, related to Fig. 5. (**a**) Gut-expressed NUC-1::mCherry in fed and starved wild-type and *spin-1,2,3* L1 worms. Bar, 5 µm. (**b**) Gut-expressed NUC-1::mCherry junctions/object in fed (*n* = 20 worms) and starved (*n* = 21 worms) wild-type and fed (*n* = 20 worms) and starved (*n* = 18 worms) *spin-1,2,3* L1 worms. F = Fed, S = Starved. Mean ± SEM. Two-way ANOVA with Tukey’s multiple comparisons. (**c**) Gut-expressed NUC-1::mCherry object lengths in fed (*n* = 393 objects from 20 worms) and starved (*n* = 471 objects from 21 worms) wild-type L1 worms and in fed (*n* = 293 objects from 20 worms) and starved (*n* = 152 objects from 18 worms) *spin-1,2,3* L1 worms. Box-and-whisker plots (minimum, 25^th^ percentile, median, 75^th^ percentile, maximum). Two-way ANOVA with Tukey’s multiple comparisons. Source data

Although the lifespan extension observed in animals with TLs is certainly intriguing, this observation is merely correlative and does not directly implicate TLs in promoting animal health and/or longevity since starvation induces multiple metabolic pathways that might contribute to this physiological phenotype. To more directly test whether TLs contribute to the pro-health effects of DR, we asked whether eliminating TLs would suppress DR-dependent lifespan extension. Given that SPIN proteins are localized to TLs, we tested whether combinatorial mutation of the *spin* paralogs would be sufficient to prevent starvation-induced TLs. Indeed, when we imaged NUC-1::mCherry in *spin-1; spin-2; spin-3* triple mutants, we found that TLs failed to form in response to starvation in this genetic background (Fig. 5a–c and Extended Data Fig. 6a–c).

**Extended Data Fig. 7:**
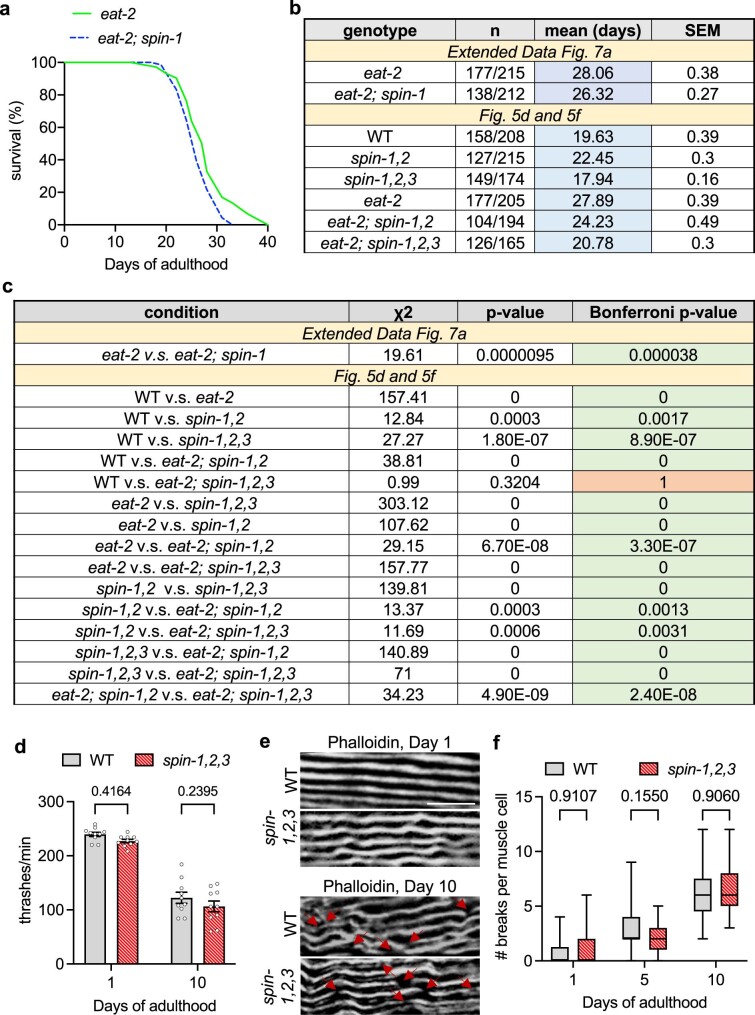
*spin-1,2,3* mutants do not exhibit negative health effects under non-DR conditions, related to Fig. 5. (**a**) Lifespan of *eat-2* and *eat-2; spin-1* mutants. (**b**) Descriptive statistics for lifespans in Fig. 5d and 5f and in Extended Data Fig. 7a. (**c**) Log-rank test results for lifespan comparisons in Fig. 5d and 5f and in Extended Data Fig. 7a. (**d**) Thrashing rate for wild-type and *spin-1,2,3* adult worms (*n* = 10 worms per condition). Mean ± SEM. Two-way ANOVA with Šídák’s multiple comparisons. (**e**) Muscle actin fibers labelled with Phalloidin in day 1 and day 10 wild-type and *spin-1,2,3* adult worms. Bar, 5 μm. (**f**) Number of breaks per muscle cell in wild-type adult worms at day 1 (*n* = 30 muscles from 12 worms), day 5 (*n* = 33 muscles from 12 worms), and day 10 (*n* = 33 muscles from 12 worms), and in *spin-1,2,3* adult worms at day 1 (*n* = 33 muscles from 12 worms), day 5 (*n* = 35 muscles from 12 worms), and day 10 (*n* = 31 muscles from 12 worms). This experiment was done in conjunction with the data presented in Fig. 7g, and the same wild-type data are presented in both graphs. Box-and-whisker plots (minimum, 25^th^ percentile, median, 75^th^ percentile, maximum). Two-way ANOVA with Šídák’s multiple comparisons. Source data

**Extended Data Fig. 8:**
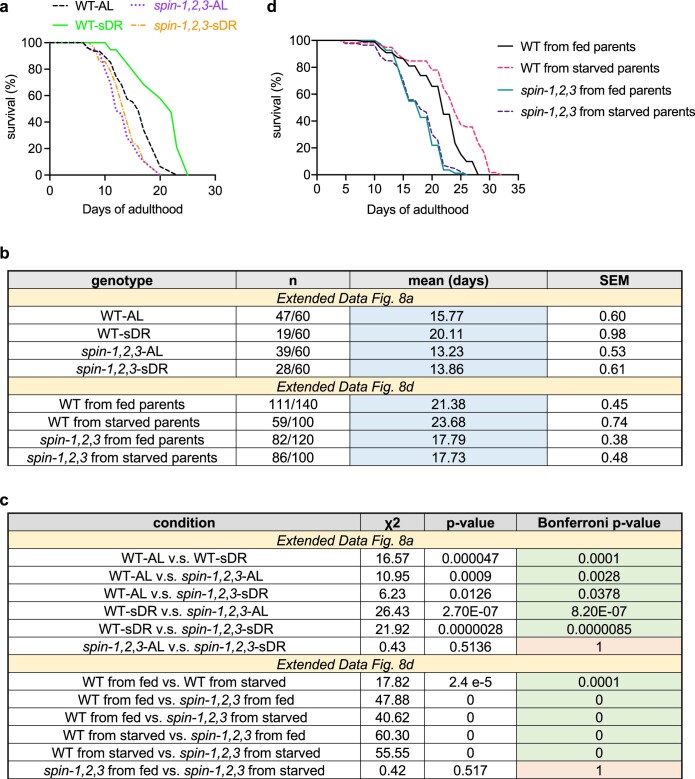
TL induction is required for sDR-dependent lifespan extension and the transgenerational effects of starvation on lifespan, related to Fig. 5. (**a**) Lifespan of wild-type and *spin-1,2,3* worms fed *ad libitum* (AL) or subjected to DR on solid medium (sDR) starting from day 4 of adulthood. (**b**) Descriptive statistics for lifespans in Extended Data Fig. 8a, d. (**c**) Log-rank test results for lifespan comparisons in Extended Data Fig. 8a, d. (**d**) Lifespan of wild-type and *spin-1,2,3* worms propagated from fed and starved parents. Source data

We then examined whether genetic mutation of *spin* genes would reduce the lifespan extension of *eat-2* mutants. Although mutation of *spin-1* alone only slightly reduced the lifespan of *eat-2* mutants (Extended Data Fig. 7a–c), we found that *eat-2* lifespan extension was more significantly reduced by double mutation of *spin-1* and *spin-2* and was further reduced back to wild type in a *spin-1; spin-2; spin-3* triple*-*mutant background (Fig. 5d and Extended Data Fig. 7b,c). This suggests that *spin* paralogs may have redundant functions. Moreover, combinatorial mutation of *spin-1*, *spin-2* and *spin-3* accelerated age-dependent mobility decline in *eat-2* mutants (Fig. 5e), further indicating that SPIN activity may be required for the full beneficial effects of DR. Importantly, analogous effects on lifespan and health were not seen in wild-type *eat-2(+)* animals with *spin* mutations. *spin-1; spin-2* double mutants did not display a reduction in lifespan relative to wild type, and *spin-1; spin-2; spin-3* triple mutants displayed only a modest reduction in lifespan and no significant differences in age-dependent mobility or muscle health under normal conditions (Fig. 5f and Extended Data Fig. 7b–f). To corroborate the effect of *spin* mutation on DR-dependent lifespan extension using another model, we performed lifespan analysis on wild-type and *spin-1; spin-2; spin-3* triple mutants under sDR conditions. We found that *spin-1; spin-2; spin-3* triple mutants, unlike wild-type animals, showed no extension to lifespan under sDR conditions (Extended Data Fig. 8a–c), confirming a requirement for SPIN protein functionality in DR-triggered longevity.

Because we had found that TLs are induced transgenerationally after a starvation event (Fig. 4b,c), we asked whether TLs would also contribute to the lifespan extension observed in the descendants of starved parents^38^. To test this, we compared the lifespan of offspring that descended from starved parents or from five generations of well-fed ancestors in wild-type and *spin* mutant backgrounds. Consistent with previous observations^38^, we found that wild-type animals that descended from starved parents lived significantly longer compared to wild-type animals that descended from well-fed generations (Extended Data Fig. 8b–d). In contrast, we observed no significant difference in the lifespans of *spin-1; spin-2; spin-3* triple mutants that came from starved parents or from well-fed ancestors (Extended Data Fig. 8b–d). Thus, TLs also support transgenerational longevity.

Lastly, we were curious whether impeding TL formation limited autophagic turnover capacity. We compared autophagic turnover at lysosomes in wild-type versus *spin* mutants by tracking SQST-1::mCherry::GFP fluorescence under fed and starved conditions. Like wild-type animals, *spin* mutants showed decreased GFP signal relative to mCherry during starvation (Fig. 5g–j). However, the amplitude of this decrease in GFP signal was smaller in *spin* mutants compared to wild-type controls; starved *spin* mutants retained higher GFP fluorescence compared to starved wild-type animals (Fig. 5g–j). These data suggest that autophagic cargo can be turned over at vesicular lysosomes to an extent, but TL induction heightens turnover above basal levels, probably contributing to longevity linked to food deprivation.

### Constitutive induction of gut TLs promotes healthy aging

**Fig. 6:**
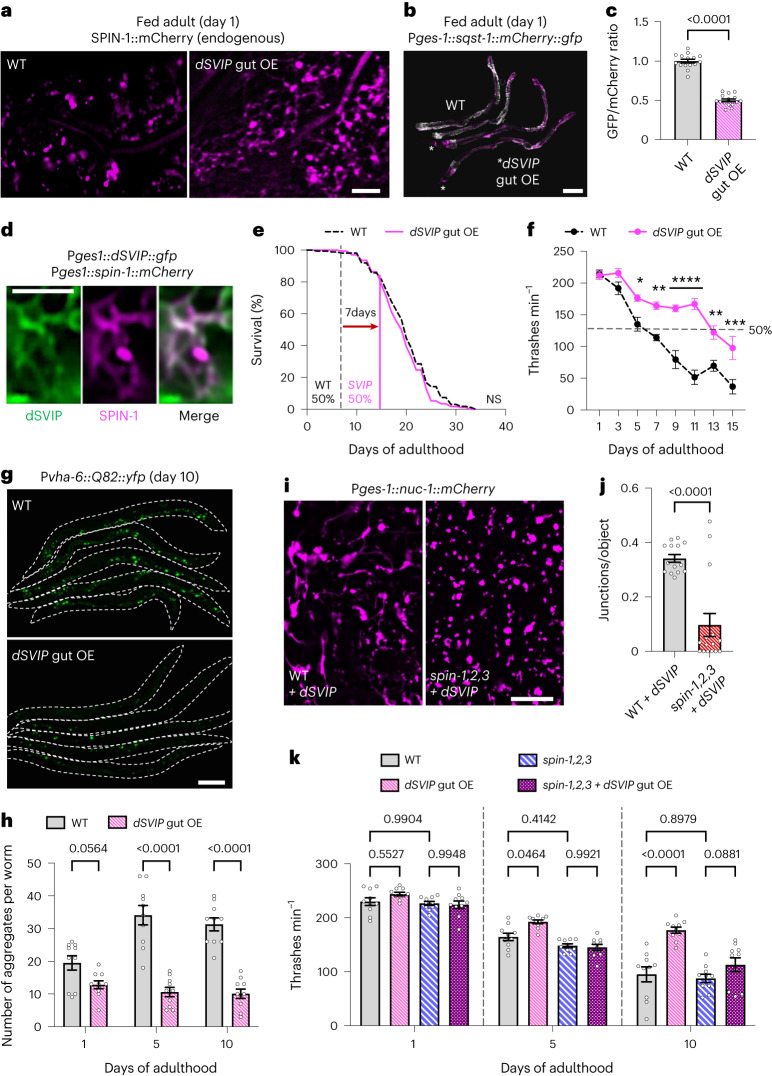
Constitutive induction of gut TLs promotes healthy aging. **a**, Endogenously tagged SPIN-1::mCherry in fed wild-type adult worms and in adult worms over-expressing *dSVIP*. Scale bar, 5 μm. **b**, Gut-expressed SQST-1::mCherry::GFP in day 1 wild-type and *dSVIP* over-expressing adult worms. Scale bar, 100 µm. **c**, GFP/mCherry ratio of gut-expressed SQST-1::mCherry::GFP in day 1 wild-type adult worms (*n* = 15 worms) and in day 1 wild-type worms over-expressing *dSVIP* (*n* = 15 worms). Mean ± s.e.m. Unpaired two-tailed Student’s *t*-test. **d**, dSVIP::GFP and SPIN-1::mCherry co-localization (*n* = 10 worms). Scale bar, 5 μm. **e**, Lifespan of wild-type and *dSVIP* over-expressing worms. Vertical lines indicate the ages at which 50% starting thrashing rate was reached. A log-rank test was used to determine statistical significance. For statistics, see Extended Data Fig. 9c,d. **f**, Thrashing rate of wild-type and *dSVIP* over-expressing adult worms (*n* = 10 worms per condition). The dashed line represents 50% of starting thrashing rate. Mean ± s.e.m. Two-way ANOVA with Šídák’s multiple comparisons (*P < 0.05, **P < 0.01, ***P < 0.001, ****P < 0.0001). **g**, Gut-expressed Q82::YFP in day 10 wild-type and *dSVIP* over-expressing adult worms (outlined). Scale bar, 100 μm. **h**, Number of gut Q82::YFP aggregates in wild-type and *dSVIP* over-expressing adult worms (*n* = 10 worms per condition). Mean ± s.e.m. Two-way ANOVA with Šídák’s multiple comparisons. **i**, Gut-expressed NUC-1::mCherry in day 1 wild-type and *spin-1,2,3* adult worms that also over-expressed *dSVIP* in the gut. Scale bar, 5 μm. **j**, Gut-expressed NUC-1::mCherry junctions/object in day 1 wild-type (*n* = 14 worms) and *spin-1,2,3* (*n* = 15 worms) adult worms that also over-expressed *dSVIP* in the gut. Mean ± s.e.m. Unpaired two-tailed Student’s *t*-test. **k**, Thrashing rate of wild-type and *spin-1,2,3* adult worms with and without *dSVIP* over-expression (*n* = 10 worms per condition). Mean ± s.e.m. Two-way ANOVA with Tukey’s multiple comparisons. OE, over-expression. Source data

**Extended Data Fig. 9:**
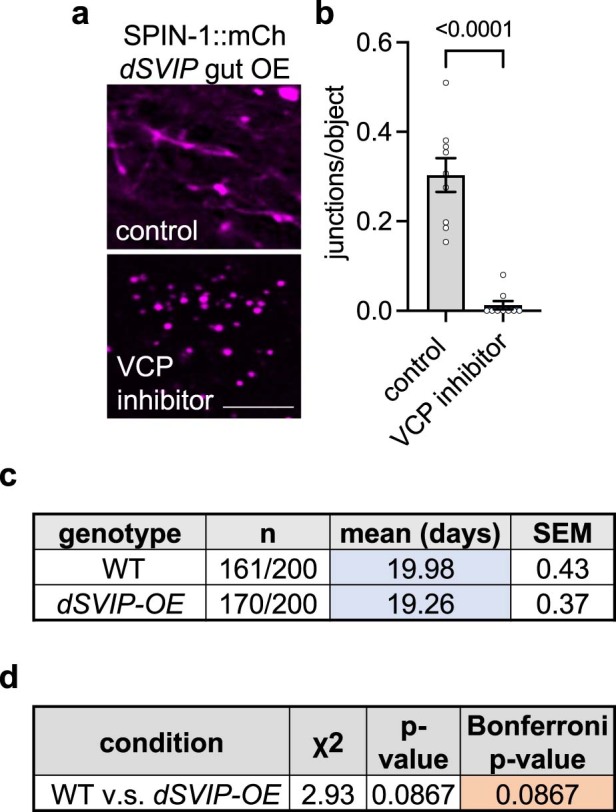
Induction of gut TLs via *dSVIP* over-expression is dependent on VCP activity and does not extend lifespan in wild-type animals, related to Fig. 6. (**a**) Endogenously-tagged SPIN-1::mCherry in day 2 adult control and VCP inhibitor-treated well-fed worms that also over-expressed *Drosophila SVIP* in the gut. Bar, 5 μm. (**b**) Endogenously-tagged SPIN-1::mCherry junctions/object (*n* = 9 worms per condition). Mean ± SEM. Unpaired two-tailed Student’s t-test. (**c**) Descriptive statistics for lifespans in Fig. 6e. (**d**) Log-rank test results for lifespan comparisons in Fig. 6e. Source data

Because we had found TLs to be required for the full lifespan extension under DR, we tested whether TL induction is sufficient to exert pro-health effects on its own. To do this, we used a genetic strategy to ectopically induce TLs in well-fed animals and examined the effect on lifespan and late-age health. Previously, we identified the small VCP-interacting protein (SVIP) as a critical TL modulator; over-expression of *SVIP* in *Drosophila* muscles was sufficient to increase TL density^19^. The *C. elegans* genome does not possess an annotated *SVIP* ortholog, so we generated a transgenic worm strain that over-expressed a codon-optimized *Drosophila SVIP* gene (*dSVIP*). Indeed, over-expression of *dSVIP* in the *C. elegans* gut was sufficient to induce gut TL formation and to support heightened autophagic activity in the gut under well-fed conditions (Fig. 6a–c). dSVIP co-localized with TLs as expected (Fig. 6d) and required the function of VCP to induce TLs (Extended Data Fig. 9a,b). Thus, SVIP has a general capacity to induce TLs in a VCP-dependent manner in multiple tissues and species.

We then explored the physiological effects of *dSVIP* over-expression in the *C. elegans* gut. Although we observed no significant effect on lifespan (Fig. 6e and Extended Data Fig. 9c,d), we noticed that *dSVIP* over-expressing worms appeared to retain their mobility later into their life. To investigate this further, we assayed the thrashing rate throughout the lifespan of control and *dSVIP* over-expressing worms. Remarkably, *dSVIP* over-expressing worms remained more active later into life (Fig. 6f and Supplementary Videos 1 and 2). In fact, the age at which *dSVIP* over-expression worms reached 50% of their starting thrashing rate was double that of wild-type worms (Fig. 6e,f). Moreover, *dSVIP* over-expression also suppressed age-related aggregation of polyQ proteins in the gut (Fig. 6g,h). Prolonged mobility and reduced protein aggregation are strong indicators of increased healthspan^51,52^; thus, while *dSVIP*-dependent TL induction in the gut did not extend the average age of mortality, TLs might promote healthier aging.

Finally, we tested whether the pro-health effects of *dSVIP* over-expression were dependent on TL induction by over-expressing *dSVIP* in *spin-1; spin-2; spin-3* triple mutants. First, we confirmed that *dSVIP* over-expression was unable to efficiently induce TLs in *spin-1; spin-2; spin-3* triple mutants by imaging NUC-1::mCherry (Fig. 6i,j). Then, we examined thrashing rates at days 1, 5 and 10 of adulthood. Importantly, *dSVIP* over-expression did not significantly improve late-age mobility in *spin-1; spin-2; spin-3* triple mutants (Fig. 6k), as it had in otherwise wild-type animals. These data are consistent with TL induction being required for the pro-health effects of *dSVIP* over-expression.

### TL induction in the gut improves muscle health

**Fig. 7:**
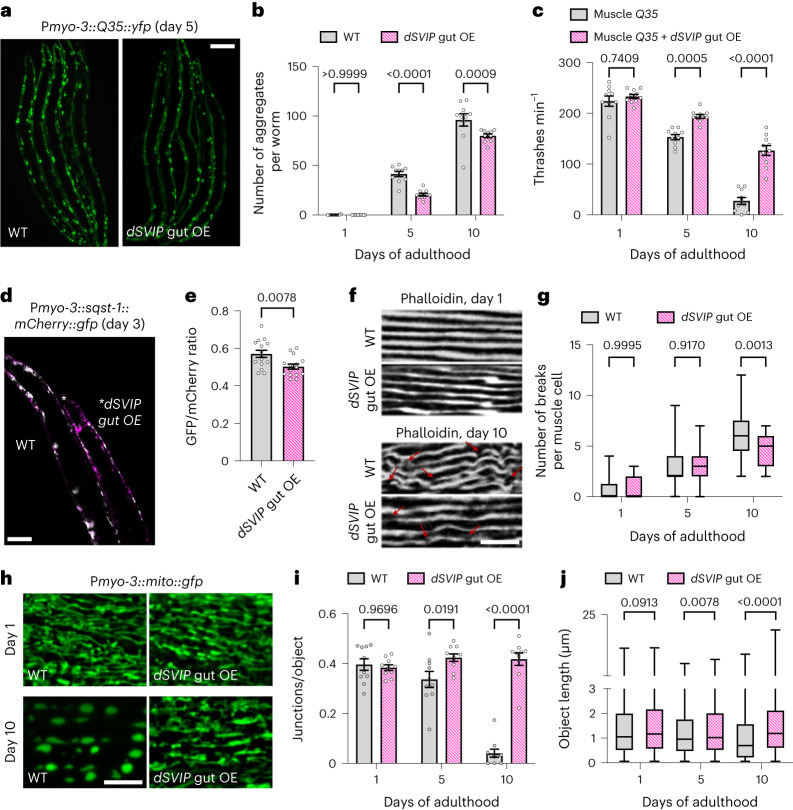
TL formation in the gut improves muscle health. **a**, Muscle-expressed Q35::YFP in day 5 wild-type and *dSVIP* over-expressing adult worms. Scale bar, 100 μm. **b**, Number of Q35::YFP aggregates in wild-type and *dSVIP* over-expressing adult worms (*n* = 10 worms per condition). Mean ± s.e.m. ANOVA with Šídák’s multiple comparisons. **c**, Thrashing rate of muscle *Q35::yfp* adult worms with and without *dSVIP* over-expression (*n* = 10 worms per condition). Mean ± s.e.m. Two-way ANOVA with Šídák’s multiple comparisons. **d**, Muscle-expressed SQST-1::mCherry::GFP in day 3 wild-type and *dSVIP* over-expressing adult worms. Scale bar, 100 μm. **e**, GFP/mCherry ratio of muscle-expressed SQST-1::mCherry::GFP in day 3 wild-type and *dSVIP* over-expressing adult worms (*n* = 15 worms per condition). Mean ± s.e.m. Unpaired two-tailed Student’s *t*-test. **f**, Muscle actin fibers labeled with Phalloidin in day 1 and day 10 wild-type and *dSVIP* over-expressing adult worms. Red arrows indicate breaks in actin fibers. Scale bar, 5 μm. **g**, Number of breaks per muscle cell in wild-type adult worms at day 1 (*n* = 30 muscles from 12 worms), day 5 (*n* = 33 muscles from 12 worms) and day 10 (*n* = 33 muscles from 12 worms), and in *dSVIP* over-expressing adult worms at day 1 (*n* = 41 muscles from 12 worms), day 5 (*n* = 41 muscles from 12 worms) and day 10 (*n* = 32 muscles from 12 worms). Box-and-whisker plots (minimum, 25th percentile, median, 75th percentile, maximum). Two-way ANOVA with Šídák’s multiple comparisons. **h**, Muscle-expressed Mito::GFP in wild-type and *dSVIP* over-expressing adult worms. Scale bar, 5 μm. **i**, Muscle-expressed Mito::GFP junctions/object in wild-type and *dSVIP* over-expressing adult worms (*n* = 10 worms per condition). Mean ± s.e.m. Two-way ANOVA with Šídák’s multiple comparisons. **j**, Muscle-expressed Mito::GFP object lengths in wild-type adult worms at day 1 (*n* = 1,308 objects from 10 worms), day 5 (*n* = 1,541 objects from 10 worms) and day 10 (*n* = 500 objects from 10 worms), and in *dSVIP* over-expressing adult worms at day 1 (*n* = 1,292 objects from 10 worms), day 5 (*n* = 776 objects from 10 worms) and day 10 (*n* = 1,582 objects from 10 worms). Box-and-whisker plots (minimum, 25th percentile, median, 75th percentile, maximum). Two-way ANOVA with Šídák’s multiple comparisons. OE, over-expression. Source data

Our observation that over-expression of *dSVIP* in the gut improved late-age mobility (Fig. 6f) suggests that TL induction in the gut might elicit systemic benefits. In particular, given the improvement to mobility, it seems likely that muscle health may be enhanced. The potential crosstalk between muscle and gut tissues is further underscored by our observation that expression of aggregation-prone polyQ proteins in muscles triggers TL induction in the gut (Fig. 3c–e). Therefore, we examined several parameters of muscle health when TLs were induced in the gut via *dSVIP* over-expression. First, we expressed aggregation-prone Q35::YFP in muscles, which produces protein aggregates in an age-dependent manner and accelerates late-age mobility impairment^53^. Remarkably, over-expression of *dSVIP* in the gut suppressed age-dependent Q35::YFP aggregation in muscle (Fig. 7a,b) and also sustained enhanced mobility with age in the muscle *Q35* animals (Fig. 7c). Consistent with suppression of protein aggregation in muscles, we also observed increased basal levels of autophagic turnover in muscles of *dSVIP* over-expressing worms (Fig. 7d,e), as had been seen in their gut (Fig. 6b,c). Finally, we found that muscle fiber integrity was maintained and that age-dependent mitochondrial fragmentation in muscle was delayed when *dSVIP* was over-expressed in the gut (Fig. 7f–j). These data demonstrate that TL induction in the gut can improve the health of other tissues through a cell non-autonomous mechanism.

### Hyperactivation of TLs can compensate for autophagy defects

**Extended Data Fig. 10:**
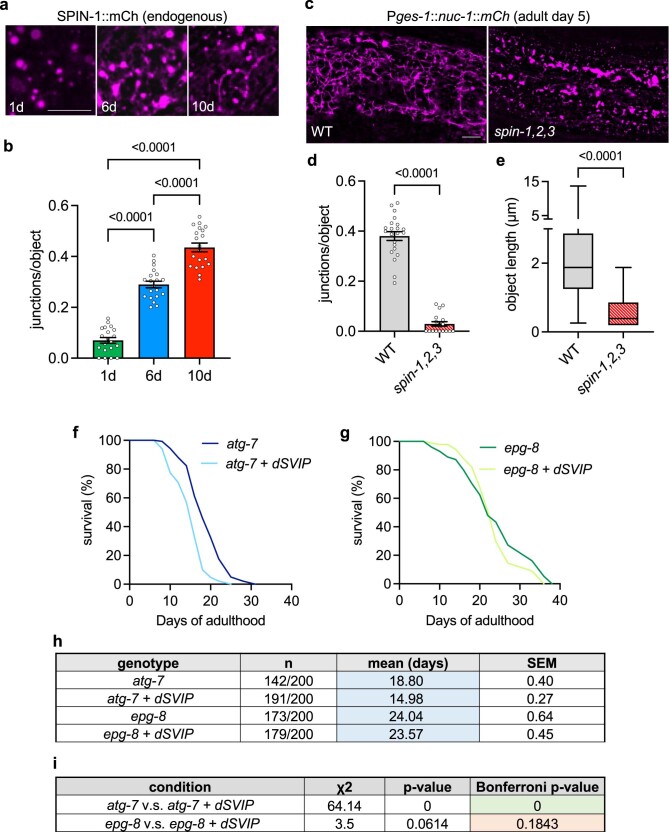
Natural aging induces TLs in the *C. elegans* gut in a SPIN-dependent manner and induction of gut TLs does not extend lifespan in hypomorphic autophagy mutants, related to Fig. 8. (**a**) Endogenously-tagged SPIN-1::mCherry worms at days 1, 6, and 10 of adulthood. Bar, 5 µm. (**b**) Endogenously-tagged SPIN-1::mCherry junctions/object (*n* = 20 worms per age). Mean ± SEM. One-way ANOVA with Tukey’s multiple comparisons. (**c**) Gut-expressed NUC-1::mCherry in day 5 wild-type and *spin-1,2,3* adult worms. Bar, 5 µm. (**d**) Gut-expressed NUC-1::mCherry junctions/object in day 5 wild-type (*n* = 22 worms) and *spin-1,2,3* (*n* = 18 worms) adult worms. Mean ± SEM. Unpaired two-tailed Student’s t-test. (**e**) Gut-expressed NUC-1::mCherry object lengths in day 5 wild-type (*n* = 2,670 objects from 22 worms) and *spin-1,2,3* (*n* = 390 objects from 18 worms) adult worms. Box-and-whisker plots (minimum, 25^th^ percentile, median, 75^th^ percentile, maximum). Unpaired two-tailed t-test with Welch’s correction. (**f, g**) Lifespans of the indicated genotypes. (**h**) Descriptive statistics for lifespans in Extended Data Fig. 10f, g. (**i**) Log-rank test results for lifespan comparisons in Extended Data Fig. 10f, g. Source data

Collectively, we have multiple lines of evidence suggesting that lysosomes respond to specific metabolic shifts and deploy TLs to meet those needs. First, TLs are triggered by nutrient deprivation or increased proteotoxic stress and, when induced, can clear autophagic cargo more efficiently compared to their vesicular counterparts. Second, TLs are not dependent on autophagosome formation but are in fact triggered in autophagy-defective mutants when proteotoxic stress is again increased due to the accumulation of uncleared autophagic cargo. In accord with these observations as well as those of others^54^, we have also found that TLs are naturally induced in a SPIN-dependent manner during aging (Extended Data Fig. 10a–e), when autophagy mechanisms are known to decline^55^. Finally, when TLs are prevented from forming under these stimuli, autophagic cargo is not cleared as efficiently and the pro-health effects are negated. These data suggest that TLs have the capacity to sense a rise in autophagic demand and to enhance cargo turnover above basal levels.

**Fig. 8:**
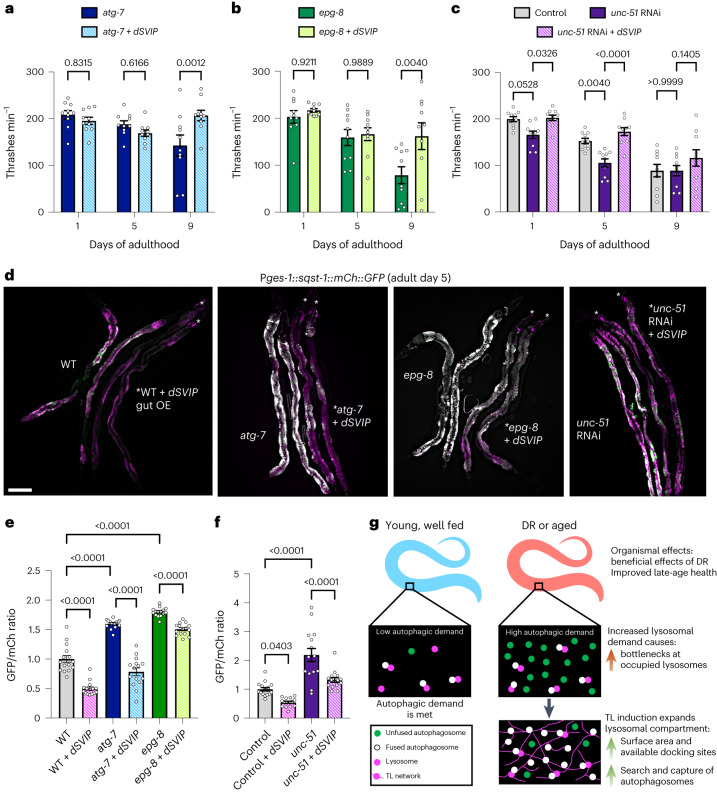
Hyperactivation of TLs can compensate for autophagy defects. **a**, Thrashing rate of *atg-7* mutants with and without *dSVIP* over-expression (*n* = 10 worms per condition). Mean ± s.e.m. Two-way ANOVA with Šídák’s multiple comparisons. **b**, Thrashing rate of *epg-8* mutants with and without *dSVIP* over-expression (*n* = 10 worms per condition). Mean ± s.e.m. Two-way ANOVA with Šídák’s multiple comparisons. **c**, Thrashing rate of wild-type adult worms fed control RNAi, wild-type adult worms fed *unc-51* RNAi, and *dSVIP* over-expressing adult worms fed *unc-51* RNAi (*n* = 10 worms per condition). Mean ± s.e.m. Two-way ANOVA with Tukey’s multiple comparisons. **d**, Gut-expressed SQST-1::mCherry::GFP in day 5 wild-type, *atg-7*, *epg-8* and *unc-51*-RNAi adult worms with and without *dSVIP* over-expression. Scale bar, 100 µm. **e**, GFP/mCherry ratio of gut-expressed SQST-1::mCherry::GFP in wild-type, *atg-7* and *epg-8* adult worms with and without *dSVIP* over-expression (*n* = 15 worms per condition). Mean ± s,e.m. One-way ANOVA with Šídák’s multiple comparisons. **f**, GFP/mCherry ratio of gut-expressed SQST-1::mCherry::GFP in control and *unc-51*-RNAi adult worms with and without *dSVIP* over-expression (*n* = 15 worms per condition). Mean ± s.e.m. One-way ANOVA with Šídák’s multiple comparisons. **g**, A summary model depicting the morphological adaptation of lysosomes during nutrient deprivation that contributes to improved organismal health. In response to higher demands for lysosomal activity during DR, lysosomes expand into a dynamic and extensive tubular network that increases the surface area, providing more docking sites for autophagosome fusion and cargo degradation. Expansion of the lysosome compartment relieves bottlenecks at occupied lysosomes and might also assist in more efficient search and capture of cargo-loaded autophagosomes. Source data

To further test this idea, we asked whether hyperactivating TLs via *dSVIP* over-expression could suppress partial defects in the autophagy system caused by gene mutation or gene knockdown. Remarkably, *dSVIP* over-expression enhanced late-age mobility, but not lifespan, in hypomorphic *atg-7* and *epg-8/atg-14* autophagy mutants (Fig. 8a,b and Extended Data Fig. 10f–i), which are defective in autophagosome formation and fusion, respectively. We also aimed to test whether this applied to animals defective in UNC-51/ATG-1. Because *unc-51* mutant animals are nearly immobile due to neurodevelopmental abnormalities caused by an autophagy-independent mechanism^34,56^, we alternatively treated animals with *unc-51* RNA interference (RNAi) starting at day 1 of adulthood to bypass the developmental defects. As in the hypomorphic autophagy mutants, *dSVIP* over-expression likewise enhanced late-age mobility of animals treated with *unc-51* RNAi (Fig. 8c). We further examined whether these improvements were associated with heightened autophagic turnover by imaging gut-driven SQST-1::mCherry::GFP. Indeed, SQST-1 turnover at lysosomes increased in *atg-7* and *epg-8* mutants, as well as in *unc-51*-RNAi animals, when *dSVIP* was over-expressed (Fig. 8d–f). Taken together, these data suggest that TL hyperactivation can produce pro-health effects even when autophagic efficiency is suboptimal, perhaps by compensating for partial loss of function in a separate branch of the autophagy machinery.

## Discussion

Dietary-based interventions have long been known to have beneficial effects on organismal health and aging. Although autophagy activity has been linked to DR-dependent benefits, we provide evidence that morphological changes to lysosomes may also have a central role in eliciting the full beneficial effects of DR. Our results demonstrate that, in some tissues, lysosomes can mobilize into highly digestive tubular networks in response to nutrient deprivation or other cues to promote organismal health and physiology (Fig. 8g). By directly quantifying cargo turnover using a newly developed SQST-1 dual-fluorescent reporter, we found that, in conditions where TLs are deployed, autophagic cargo is turned over more proficiently when TLs are present compared to when lysosomes can only exist in vesicular form. Moreover, constitutive activation of TLs in the gut further enhances autophagic cargo clearance in wild-type animals above basal levels. These findings add to growing evidence indicating that lysosome activity may not be purely defined by acidity or enzyme composition per se. While lumenal acidity is certainly important for hydrolase function, greater acidity does not necessarily equate with higher activity; in some cases, the most acidic lysosomes are in fact the least degradative^57^, suggesting other mechanisms may be needed to hyper-stimulate lysosomes when needed. Our data indicate that transformation into expansive, tubular networks could provide an independent, perhaps superior means to heighten lysosome proficiency. We propose a model in which expansion into a tubular network establishes a larger dynamic surface area that can actively participate in ‘search and capture’ of molecular cargo^26^ and increase lysosomal platform coverage within a cell (Fig. 8g).

If TLs support organismal health, why might TLs be deployed in some tissues only at certain times rather than constitutively? One possibility is that maintenance of such a large dynamic network is energetically prohibitive under basal conditions when autophagic demand is minimal. Thus, TLs could be restrained and only deployed when large pools of cargo need to be degraded en masse. This appears to be the case during starvation^17^ but may also apply to other scenarios requiring a recalibration of cellular homeostasis, such as during adverse metabolic conditions or major developmental transitions^15,16,18^. Indeed, TL induction has been reported to be induced by *C. elegans* molting^15^ and by *Drosophila* metamorphosis^16^. Interestingly, our findings also suggest that the mere presence of uncleared cargo, rather than its delivery to lysosomes, may be sufficient to trigger this shift in lysosome morphology, perhaps serving as a preemptive signal that communicates a need for more robust recycling mechanisms.

Molecularly, how TLs are generated from vesicular lysosomes remains an open question. Our observations that SPIN proteins are required for TL induction in multiple biological contexts (Fig. 5a–c and Extended Data Figs. 6a–c and 10c–e) and generally increase in abundance with TL induction (Fig. 1d–g and Extended Data Figs. 2g,h and 4c–f,i,j; ref. ^18^) hint that expression levels of *spin* genes may be one key determinant of TL formation. However, other molecular factors probably coordinate with *spin* to induce TLs in *C. elegans* as *spin-1* over-expression alone is not sufficient to induce TLs (Fig. 4b; ref. ^17^). In addition to SPIN proteins, other factors required to promote expansion of a dynamic lysosomal network could include (1) factors that can supply sources of additional membrane material, (2) membrane-associated proteins and enzymes that can aid in membrane fusion and scission events, and (3) molecular motors that can support its dynamicity. Performing screens to identify such factors would certainly be informative to better understand the molecular underpinnings of TL biology.

Importantly, applying knowledge of TL induction strategies may suggest new therapeutic avenues for treating diseases based on lysosome and/or autophagy dysfunction. In both flies^19^ and worms, the protein SVIP appears to provide one entry point to potentially link TL stimulation to improved late-age health, even when other components of the autophagy–lysosome system are pharmacologically or genetically impaired. By driving TL expansion, heightened SVIP activity may buffer against other autophagy defects by enhancing robustness of the larger lysosome network. Indeed, our results indicate that TL induction via *dSVIP* over-expression is sufficient to partially rescue autophagic flux in scenarios where autophagosome formation and/or fusion is impaired. Because SVIP is a very small protein, composed of 82 amino acids in flies and 77 amino acids in mammals, it may even be adaptable as an exogenous therapeutic peptide once minimal active fragments are identified. Remarkably, stimulating TLs constitutively in the gut via *dSVIP* over-expression in this tissue alone also improves autophagic turnover and cellular health of a separate tissue. ‘Tele-proteostasis’ mechanisms, whereby enhancing proteostasis in one tissue can improve proteostasis in other tissues, have been reported previously^58–60^; thus, TL induction may represent another means of inducing ‘tele-proteostasis’ to boost systemic health. Finally, we found that inducing TLs constitutively beginning at young adulthood was sufficient to improve late-age health but did not extend total lifespan. Although this was an unexpected finding, there is growing evidence indicating that health and lifespan are not always strictly synched. For example, although *daf-2* insulin signaling mutants are long-lived and show improved physiological performance when compared to chronologically age-matched wild-type animals, they spend a disproportionately longer time in a decrepit state in the later period of their life^61^. Thus, their total healthspan does not scale precisely with their lifespan, and their ability to survive longer may be due to an increased ability to resist infection-related death^62^. As a corollary, there are examples of long-lived animals that in fact age faster at the molecular level^63^. Thus, healthspan and lifespan can be uncoupled, and our studies present a unique example where healthspan is increased while lifespan is unaffected. Importantly, an intervention that reduces an animal’s period of morbidity may be preferable, both personally and economically, compared to one that only extends maximal lifespan^64^. Accordingly, devising strategies that can extend the period of healthy life may be more practically advantageous.

In sum, our findings provide strong evidence that TL induction promotes healthy aging and could be a viable target for age-related disease interventions. Our observations also indicate that lysosome tubulation is not always separable from autophagy; in some cases, such as DR, lysosome tubulation is in fact intimately linked to autophagic activity, even supporting more robust cargo turnover. Further studying this unique population of autophagic lysosomes is likely to reveal additional aspects of cell biology previously missed and to suggest potential therapeutic applications relevant to animal aging and longevity.

## Methods

### Strain generation

Supplementary Table 1 provides a complete list of strains used in this study. Endogenously tagged *spin-1::mCherry*, *spin-2::mCherry* and *spin-3::mCherry* were generated by In Vivo Biosystems using CRISPR technology. All other strains used in this study were generated using standard genetic crosses or microinjection^65^. For genetic crosses, transgenes expressing fluorescent proteins were tracked by stereomicroscopy, and gene deletions and mutations were verified by nested polymerase chain reactions (PCRs) and/or sequencing. For microinjection, constructs were injected individually or in combination into the gonad of adult hermaphrodites, each at a concentration of 25 ng µl^−1^. Integration of transgenes was achieved using ultraviolet irradiation, followed by more than five generations of outcrossing. The following transgenes were generated in-lab:

P *ges-1::sqst-1::mCherry::gfp::unc-54* UTR and P *myo-3::sqst-1::mCherry::gfp::unc-54* UTR

*sqst-1* was PCR-amplified from *C. elegans* genomic DNA with the stop codon replaced with an EcoRI site using the following primers: forward, 5′-GGG GAC AAG TTT GTA CAA AAA AGC AGG CTA TGG CTG CTG CAT CAT CCG CTC CTC-3′; reverse, 5′-GGG GAC CAC TTT GTA CAA GAA AGC TGG GTG AAT TCG TGA AGA AGC GCC TGA AGA C-3′. This *sqst-1* sequence was then cloned into the pDONR221 Gateway entry vector using BP clonase (ThermoFisher), and the insert was verified by DNA sequencing. An *mCherry::gfp* coding sequence was PCR-amplified (from a lab stock plasmid^17^) with a stop codon after the *gfp* coding sequence and appropriate 5′ and 3′ overlap sequences using the following primers: forward, 5′-CTC GAG ATG TGT CTT CAG GCG CTT CTT CAC GAA TTC ATG GTC TCA AAG GGT GAA GAA GAT-3′; reverse, 5′-GCC AAC TTT GTA CAA GAA AGC TGG GTG AAT TCT TAT AGT TCA TCC ATG CCA TGT GTA ATC-3′. The pDONR221 *sqst-1* plasmid was linearized by digesting with EcoRI and the *mCherry::gfp* PCR product was inserted at the C-terminus of *sqst-1* using a Gibson-assembly reaction (ThermoFisher). pDONR221 *sqst-1::mCherry::gfp* was sequence-verified and then combined with lab-stock plasmids pDONR P4-P1r P*ges-1* and pDONR P2R-P3 *unc-54* 3′ untranslated region (UTR) into the pDEST R4-R3 Gateway destination vector using LR clonase (ThermoFisher). pDONR221 *sqst-1::mCherry::gfp* was also combined with pDONR P4-P1r P*myo-3* and pDONR P2R-P3 *unc-54* 3′ UTR into the pDEST R4-R3 Gateway destination vector to generate P*myo-3::sqst-1::mCherry::gfp::unc-54* UTR.

P *ges-1::nuc-1::mCherry::unc-54 UTR*

*nuc-1* was PCR-amplified from *C. elegans* genomic DNA with the stop codon replaced with a SacI site using the following primers: forward, 5′-GGG GAC AAG TTT GTA CAA AAA AGC AGG CTA TGG GCT TGT CTC CTG CCG CTG TGC-3′; reverse, 5′-GGG GAC CAC TTT GTA CAA GAA AGC TGG GT GAG CTC TGC ACA ATT ATT TTG GGT TGC AAT T-3′. This *nuc-1* sequence was then cloned into the pDONR221 Gateway entry vector using BP clonase (ThermoFisher), and the insert was verified by DNA sequencing. An *mCherry* coding sequence was PCR-amplified (from a lab stock plasmid^17^) with a stop codon and appropriate overlap sequences using the following primers: forward, 5′-GTA ATT GCA ACC CAA AAT AAT TGT GCA GAG CTC ATG GTC TCA AAG GGT GAA GAA GAT AAC-3′; reverse, 5′-GCC AAC TTT GTA CAA GAA AGC TGG GTG AGC TCT TAC TTA TAC AAT TCA TCC ATG CCA C-3′. The pDONR221 *nuc-1* plasmid was linearized by digesting with SacI, and the *mCherry* PCR product was then inserted at the C-terminus of *nuc-1* using a Gibson-assembly reaction (ThermoFisher). pDONR221 *nuc-1::mCherry* was sequence-verified and then combined with lab-stock plasmids pDONR P4-P1r P*ges-1* and pDONR P2R-P3 *unc-54* 3′ UTR into the pDEST R4-R3 Gateway destination vector using LR clonase (ThermoFisher).

P *ges-1::dSVIP::unc-54 UTR* and P *ges-1::dSVIP::gfp::unc-54 UTR*

The coding sequence for *Drosophila SVIP* was codon-optimized for *C. elegans* expression using the *C. elegans* Codon Adaptor^66^. This sequence was then synthesized as a gBlock by Integrated DNA Technologies and PCR-amplified using the following primers: forward, 5′-GGG GAC AAG TTT GTA CAA AAA AGC AGG CTC AAA AAA AAA TGG GAG CCT GTT TAT CAT GTT GC-3′; reverse, 5′-GGG GAC CAC TTT GTA CAA GAA AGC TGG GTT TAA GAG GTT TGC CAA CGA AGG TTA G-3′. This *dSVIP* sequence was then cloned into the pDONR221 Gateway entry vector using BP clonase (ThermoFisher), and the insert was verified by DNA sequencing. To generate a GFP-tagged version, a Gibson-assembly reaction (ThermoFisher) was performed to insert a GFP coding sequence before the *dSVIP* stop codon. The GFP coding sequence was PCR-amplified (from a lab stock plasmid^17^) using the following primers: forward, 5′-ATG AGT AAA GGA GAA GAA CTT TTC AC-3′; reverse, 5′-CTA TTT GTA TAG TTC ATC CAT GCC ATG-3′. The plasmid was PCR-amplified as a linear piece of DNA with appropriate overlaps using the following primers: forward, 5′-CAT GGC ATG GAT GAA CTA TAC AAA TAG ACC CAG CTT TCT TGT ACA AAG TTG-3′; reverse, 5′-GTG AAA AGT TCT TCT CCT TTA CTC ATA GAG GTT TGC CAA CGA AGG TTA GAT TG-3′. Following Gibson assembly, the full insert was again verified by DNA sequencing. Ultimately, pDONR221 *dSVIP* and pDONR221 *dSVIP::gfp* were each combined with lab-stock plasmids pDONR P4-P1r P*ges-1* and pDONR P2R-P3 *unc-54* 3′ UTR into the pDEST R4-R3 Gateway destination vector using LR clonase (ThermoFisher).

### Animal maintenance

For all experiments using invertebrate *C. elegans*, hermaphroditic animals were used. Ages of adult animals are specifically defined in figures and figure legends for age-specific studies and lifespan analyses. Supplementary Table 1 provides a list of all *C. elegans* strains used in this study. The invertebrate fly *Drosophila melanogaster* was also used to assess conservation of TL induction upon starvation. Third instar larvae were used for all *Drosophila* experiments, and animals were selected irrespective of sex.

Worms were raised at 20 °C on nematode growth medium (NGM) agar (51.3 mM NaCl, 0.25% peptone, 1.7% agar, 1 mM CaCl_2_, 1 mM MgSO_4_, 25 mM KPO_4_ and 12.9 µM cholesterol, pH 6.0). Fed worms were maintained on NGM agar plates that had been seeded with *Escherichia coli* OP50 bacteria. Synchronous populations of worms were obtained by bleaching young-adult hermaphrodites. Briefly, adult hermaphrodites were vortexed in 1 ml bleaching solution (0.5 M NaOH and 20% bleach) for 5 min to isolate eggs, and eggs were then washed three times in M9 buffer (22 mM KH_2_PO_4_, 42 mM Na_2_HPO_4_, 85.5 mM NaCl and 1 mM MgSO_4_) before plating. To obtain starved L1 animals, bleached eggs were spotted on NGM agar that lacked OP50 bacteria, and plates were maintained at 20 °C for 18–24 h before imaging, except where otherwise noted. For aging experiments, synchronous populations of animals were established by bleaching adult hermaphrodites or by synchronous egg-laying. In all aging experiments excluding lifespan analyses (see below), adult worms were picked onto fresh OP50-seeded NGM plates every 1–2 days to maintain adults separate from progeny.

Fly stocks were raised at 25 °C with 12:12 h light:dark cycles and fed on a standard cornmeal molasses medium. UAS-*Spin-RFP* and *esg*-GAL4 were obtained from the Bloomington *Drosophila* Stock Center.

### RNAi experiments

RNAi clones were generated for *let-363, daf-15* and *ash-2* using Gibson cloning to insert gene fragments into the cut PstI site of the L4440 vector. The *let-363* gene fragment was PCR-amplified from *C. elegans* genomic DNA using the following primers: forward, 5′-CCA CGT GAC GCG TGG ATC CCC CGG GCT GCA ATG CTC CAA CAA CAC GGA ATT AGT TTT C-3′; reverse, 5′-CGG TAT CGA TAA GCT TGA TAT CGA ATT CCG AAT GCT GTC GGT GTG GCC AGT GCG AGC TC-3′. The *daf-15* gene fragment was PCR-amplified from *C. elegans* genomic DNA using the following primers: forward, 5′-CCA CGT GAC GCG TGG ATC CCC CGG GCT GCA ATG GAA GAG GAT AGG AGT ATA ACA CCG-3′; reverse, 5′-CGG TAT CGA TAA GCT TGA TAT CGA ATT CCG CCA GGA TAT TTT CTG ATC TTC TCC AAA TC-3′. The *ash-2* gene fragment was PCR-amplified from *C. elegans* genomic DNA using the following primers: forward, 5′-CCA CGT GAC GCG TGG ATC CCC CGG GCT GCA ATG AGA AGC TCA AAA GGA GGT CGG GGA CG-3′; reverse, 5′-CGG TAT CGA TAA GCT TGA TAT CGA ATT CCC ACC GGA GCC TCG GCG TGC CGG CGT TTC G-3′. The *unc-51*, *set-2* and *wdr-5* RNAi clones were obtained from the Julie Ahringer RNAi collection^67^. All clones were verified by DNA sequencing. For RNAi experiments, synchronous populations of animals were grown on OP50-seeded NGM plates until late L4 or day 1 of adulthood, at which time they were transferred to RNAi plates (NGM plus 100 ng µl^−1^ carbenicillin and 1 mM isopropyl β-d-1-thiogalactopyranoside) that had been seeded with bacteria expressing the relevant RNAi clone. An empty L4440 vector was used as a negative control.

### sDR treatment

sDR treatment was adapted from a protocol described previously^22^. Briefly, to make sDR NGM plates, an overnight saturated OP50 culture was diluted 1:1,000 in S-medium (1 M potassium citrate pH 6 and 1% trace metals solution (5 mM disodium ethylenediaminetetraacetic acid, 2.5 mM FeSO_4_•7 H_2_O, 1 mM MnCl_2_•4 H_2_O, 1 mM ZnSO_4_•7 H_2_O, 0.2 mM CuSO_4_•5 H_2_O, 1 M CaCl_2_ and 1 M MgSO_4_) in S-basal (100 mM NaCl, 6 mM K_2_HPO_4_, 44 mM KH_2_PO_4_ and 13 mM cholesterol)) to prevent further bacterial growth, and 150 μl of the dilution was seeded on to 35 mm NGM plates. For imaging, worms were transferred to sDR NGM plates at the L4 stage and imaged on day 2 of adulthood. For lifespan analyses, synchronous populations of L4 worms were transferred to NGM plates seeded with concentrated OP50 bacteria until day 4 of adulthood, at which point worms were transferred to sDR NGM plates and mortality was scored every alternate day until all worms died. Throughout all experiments using sDR NGM plates, adult worms were transferred to freshly seeded sDR NGM plates every day.

### VCP inhibitor treatment

A 10 μM stock solution of the VCP inhibitor CB5083 (MedChem Express, cat. no. HY-12861/CS-5405) was prepared in dimethyl sulfoxide (DMSO) and diluted to a final working concentration of 1 μM in M9 buffer. Three-hundred microlitres of the working stock was directly spotted onto NGM plates that were previously seeded with OP50 bacteria. For control plates, DMSO was diluted 1:10 in M9 buffer, and 300 μl was directly spotted onto NGM plates that were previously seeded with OP50 bacteria. Worms were synchronized by bleaching, and late L4 or day 1 adults were transferred to control (DMSO) or CB5083 plates. Imaging was performed 2 days later.

### Paraquat treatment

For Paraquat treatment, a 1 M stock solution of Paraquat (Acros Organics, AC22732) was prepared in water and diluted to a final concentration of 0.25 mM (low dose) or 5 mM (high dose) in NGM agar and in the OP50 bacteria seeded onto the plates. Worms were synchronized by bleaching*,* and late L4 or day 1 adults were transferred to control or Paraquat plates. Imaging was performed 1–2 days after plating on control or Paraquat plates.

### Lifespan analysis

Synchronous populations of animals were transferred as late L4s to NGM plates seeded with OP50 bacteria. Plates were previously spotted with 5 mM FUdR (Acros Organics), unless otherwise noted, to prevent progeny production. Animals that exploded, bagged or crawled off plates were censored during analysis. Lifespans were analyzed using OASIS 2 software^68^, and statistical significance was assessed using a log-rank test.

For TL sorting lifespans, synchronized eggs were seeded onto NGM agar with no food source. After 5 days, starved L1-arrested worms were transferred to food, and worm populations were continuously fed for two generations. Late L4 F_2_ progeny from starved grandparents were sorted under a fluorescence stereomicroscope and separated into two populations based on visible presence or absence of TLs. Lifespans of the two populations were analyzed using the method described above.

### Thrashing assay

Synchronous populations of animals were transferred as late L4s to NGM plates seeded with OP50 bacteria with no FUdR. Worms were transferred to fresh plates every 1–2 days to separate adults from their progeny. To score thrashing rates, individual worms were transferred into a drop of M9 buffer on an NGM plate, and the number of thrashes were counted in a 1 min period.

### Microscopy

For *C. elegans* whole animal imaging, 4% agarose (Fisher Bioreagents) pads were dried on a Kimwipe (Kimtech) and then placed on top of a Gold Seal glass microscope slide (ThermoFisher Scientific). A small volume of 2 mM levamisole (Acros Organics) was spotted on the agarose pad. Worms were transferred to the levamisole spot, and a glass cover slip (Fisher Scientific) was placed on top to complete the mounting. For *Drosophila* live-tissue imaging, salivary glands were dissected in O’Dowd’s saline buffer^69^ without calcium (101 mM NaCl, 4 mM MgCl_2_, 3 mM KCl, 5 mM glucose, 1.25 mM NaH_2_PO_4_, and 20.7 mM NaHCO_3_, pH 7.2) and mounted on a Gold Seal^TM^ glass microscope slide (ThermoFisher Scientific) with saline and a glass cover slip (Fisher Scientific). Live-animal or live-tissue fluorescence microscopy was performed using a Leica DMi8 THUNDER imager, equipped with 10X (NA 0.32), 40X (NA 1.30), and 100X (NA 1.40) objectives and GFP and Texas Red filter sets.

### Phalloidin staining

Age-synchronized worms were stained with Alexa Fluor 568 Phalloidin (Invitrogen, A12380), as previously described^70^. Briefly, worms were washed in M9 buffer, snap-frozen in liquid nitrogen, permeabilized by chilled acetone, stained with 2.5 U Alexa Fluor 568 Phalloidin, and washed in M9 before mounting. Three microlitres of worm suspension was transferred to a 4% agarose pad and imaged using an exposure time of 200 ms.

### Image analysis

Images were processed using LAS X software (Leica) and FIJI/ImageJ (National Institutes of Health, NIH). Lysosome networks were analyzed using ‘Skeleton’ analysis plugins in FIJI. Briefly, images were converted to binary 8-bit images and then to skeleton images using the ‘Skeletonize’ plugin. Skeleton images were then quantified using the ‘Analyze Skeleton’ plugin. Number of objects, number of junctions and object lengths were scored. An ‘object’ is defined by the Analyze Skeleton plugin as a branch connecting two endpoints, an endpoint and junction, or two junctions. Junctions/object was used as a parameter to quantify network integrity.

For analyzing fluorescence intensity, the tissue of interest was outlined using the free-draw tool in FIJI/ImageJ, and average fluorescence intensity of the outlined area was measured. For all SPIN-1::mCherry fluorescence intensity experiments, 50% laser intensity, 300 ms exposure time and 100% Fluorescence Intensity Manager settings were used. For SQST-1::mCherry::GFP fluorescence ratio experiments, 20% laser intensity, 200 ms exposure time and 100% Fluorescence Intensity Manager settings were used.

### Statistics and reproducibility

Data were statistically analyzed using GraphPad Prism. For all experiments, data distribution was assumed to be normal, but this was not formally tested. For two sample comparisons, an unpaired two-tailed *t*-test was used to determine significance (*α* = 0.05). For three or more samples, a one-way analysis of variance (ANOVA) with Dunnett’s, Tukey’s or Šídák’s multiple comparisons was used to determine significance (*α* = 0.05). For grouped comparisons, a two-way ANOVA with Šídák’s multiple comparisons was used to determine significance (*α* = 0.05). *P* values are indicated on bar graphs and box-and-whisker plots. For box-and-whisker plots, horizontal lines inside boxes indicate medians, box edges represent 25th and 75th percentiles, and whiskers extend to minima and maxima. Statistical significance of lifespan data was determined using a log-rank test, and statistics for lifespan data are provided in Extended Data and in source data files.

For all experiments, no statistical methods were used to predetermine sample size, but our sample sizes are similar to those reported in previous literature^17,19^. For lifespan analyses, larger beginning sample sizes (>60 animals) were used to ensure sufficient statistical power after eliminating censored animals. The only data excluded from analysis were animals censored during lifespans. Animals that crawled off plates or exploded were censored, as is common practice. Data were replicated successfully for all experiments. Replicates are noted in figures and figure legends.

No randomization method was used to allocate animals to experimental groups. The investigators were not blinded during data collection or analyses. Blinding during data collection was not possible for most of these studies since the experimental conditions caused noticeable phenotypic differences among the groups (old versus young, fed versus starved, fluorescent marker differences, and so on). Blinding during data analysis was not relevant in most cases because automated analysis software was used uniformly to quantify microscopy.

### Reporting summary

Further information on research design is available in the Nature Portfolio Reporting Summary linked to this article.

## Supplementary information

Reporting Summary

Supplementary Table 1 *C. elegans* strains used in this study.

Supplementary Video 1 Representative movie demonstrating thrashing rates of wild-type and *dSVIP* gut over-expression worms (SVIP-OE) at day 7 of adulthood.

Supplementary Video 2 Representative movie demonstrating thrashing rates of wild-type and *dSVIP* gut over-expression worms (SVIP-OE) at day 10 of adulthood.

## Data Availability

All data are available in the main text, in the supplementary materials, or in the source data files. Additional information on data sources is available upon request from the corresponding authors. All unique materials used in the study are available from the authors or from commercially available sources. This study did not use or generate any data code.

## Source data

Source Data Fig. 1 Statistical source data for Fig. 1.
Source Data Fig. 2 Statistical source data for Fig. 2.
Source Data Fig. 3 Statistical source data for Fig. 3.
Source Data Fig. 4 Statistical source data for Fig. 4.
Source Data Fig. 5 Statistical source data for Fig. 5.
Source Data Fig. 6 Statistical source data for Fig. 6.
Source Data Fig. 7 Statistical source data for Fig. 7.
Source Data Fig. 8 Statistical source data for Fig. 8.
Source Data Extended Data Fig. 2 Statistical source data for Extended Data Fig. 2.
Source Data Extended Data Fig. 3 Statistical source data for Extended Data Fig. 3.
Source Data Extended Data Fig. 4 Statistical source data for Extended Data Fig. 4.
Source Data Extended Data Fig. 5 Statistical source data for Extended Data Fig. 5.
Source Data Extended Data Fig. 6 Statistical source data for Extended Data Fig. 6.
Source Data Extended Data Fig. 7 Statistical source data for Extended Data Fig. 7.
Source Data Extended Data Fig. 8 Statistical source data for Extended Data Fig. 8.
Source Data Extended Data Fig. 9 Statistical source data for Extended Data Fig. 9.
Source Data Extended Data Fig. 10 Statistical source data for Extended Data Fig. 10.
