# Supplementary material for: Tubular lysosome induction couples animal starvation to healthy aging
Source: Nat Aging. 2023 Aug 14;3(9):1091–106. doi: 10.1038/s43587-023-00470-6 (PMC10501908; doi:10.1038/s43587-023-00470-6)
Supplement: Supplementary file 1 — Reporting Summary [file 43587_2023_470_MOESM1_ESM.pdf]

nature portfolio

Corresponding author(s): Adam Bohnert & Alyssa Johnson

Last updated by author(s): 7/11/2023

Reporting Summary

Nature Portfolio wishes to improve the reproducibility of the work that we publish. This form provides structure for consistency and transparency in reporting. For further information on Nature Portfolio policies, see our Editorial Policies and the Editorial Policy Checklist.

Statistics

For all statistical analyses, confirm that the following items are present in the figure legend, table legend, main text, or Methods section.

n/a	Confirmed	
☐	☒	The exact sample size (*n*) for each experimental group/condition, given as a discrete number and unit of measurement
☐	☒	A statement on whether measurements were taken from distinct samples or whether the same sample was measured repeatedly
☐	☒	The statistical test(s) used AND whether they are one- or two-sided<br>*Only common tests should be described solely by name; describe more complex techniques in the Methods section.*
☒	☐	A description of all covariates tested
☐	☒	A description of any assumptions or corrections, such as tests of normality and adjustment for multiple comparisons
☐	☒	A full description of the statistical parameters including central tendency (e.g. means) or other basic estimates (e.g. regression coefficient) AND variation (e.g. standard deviation) or associated estimates of uncertainty (e.g. confidence intervals)
☐	☒	For null hypothesis testing, the test statistic (e.g. *F*, *t*, *r*) with confidence intervals, effect sizes, degrees of freedom and *P* value noted<br>*Give P values as exact values whenever suitable.*
☒	☐	For Bayesian analysis, information on the choice of priors and Markov chain Monte Carlo settings
☒	☐	For hierarchical and complex designs, identification of the appropriate level for tests and full reporting of outcomes
☒	☐	Estimates of effect sizes (e.g. Cohen's *d*, Pearson's *r*), indicating how they were calculated

Our web collection on statistics for biologists contains articles on many of the points above.

Software and code

Policy information about availability of computer code

Data collection	Live-animal fluorescence microscopy was performed using a Leica DMi8 THUNDER imager, equipped with 10X (NA 0.32), 40X (NA 1.30), and 100X (NA 1.40) objectives and GFP and Texas Red filter sets. Additional information on this imaging system is provided in the Methods.
Data analysis	Images were processed using LAS X software (Leica) and FIJI/ImageJ v2.9.0/1.53t (NIH). Lysosome networks were analyzed using "Skeleton" analysis plugins in FIJI/ImageJ v2.9.0/1.53t (NIH). Data were statistically analyzed using GraphPad Prism v9. For two sample comparisons with a single variable, an unpaired t-test was used to determine significance (a=0.05). For three or more samples with a single variable, a one-way ANOVA with Dunnett's or Sidak's multiple comparisons was used to determine significance (a=0.05). For samples in which two independent variables were assessed, a two-way ANOVA with Tukey's multiple comparisons was used. Statistical significance of lifespan data was determined using a Log-rank test (OASIS-2 software). C. elegans codon adapter (https://worm.mpi-cbg.de/codons/cgi-bin/optimize.py) was used for codon optimization.

For manuscripts utilizing custom algorithms or software that are central to the research but not yet described in published literature, software must be made available to editors and reviewers. We strongly encourage code deposition in a community repository (e.g. GitHub). See the Nature Portfolio guidelines for submitting code & software for further information.

Data

Policy information about availability of data

All manuscripts must include a data availability statement. This statement should provide the following information, where applicable:

- Accession codes, unique identifiers, or web links for publicly available datasets
- A description of any restrictions on data availability
- For clinical datasets or third party data, please ensure that the statement adheres to our policy

All data are available in the main text, in the supplementary materials, or in the source data files. Additional information on data sources is available upon request from the corresponding authors. All unique materials used in the study are available from the authors or from commercially available sources. This study did not use or generate any data code.

Research involving human participants, their data, or biological material

Policy information about studies with human participants or human data. See also policy information about sex, gender (identity/presentation), and sexual orientation and race, ethnicity and racism.

Reporting on sex and gender	No human subjects were used in this study.
Reporting on race, ethnicity, or other socially relevant groupings	No human subjects were used in this study.
Population characteristics	n/a
Recruitment	n/a
Ethics oversight	n/a

Note that full information on the approval of the study protocol must also be provided in the manuscript.

Field-specific reporting

Please select the one below that is the best fit for your research. If you are not sure, read the appropriate sections before making your selection.

☒ Life sciences ☐ Behavioural & social sciences ☐ Ecological, evolutionary & environmental sciences

For a reference copy of the document with all sections, see nature.com/documents/nr-reporting-summary-flat.pdf

Life sciences study design

All studies must disclose on these points even when the disclosure is negative.

Sample size	No statistical method was used to predetermine sample size. Sample sizes were chosen based upon preliminary data and evidence from published literature (PMID: 33479240, 34689720, 35620964). For lifespan analyses, larger beginning sample sizes (>60 animals) were used to ensure sufficient statistical power after eliminating censored animals.
Data exclusions	The only data excluded from analysis were animals censored during lifespans. Animals that crawled off plates or exploded were censored, as is common practice.
Replication	Data were replicated successfully for all experiments. Replicates are noted in figures and figure legends.
Randomization	No randomization method was used to allocate animals to experimental groups.
Blinding	The investigators were not blinded during data collection or analyses. Blinding during data collection was not possible for the majority of these studies since the experimental conditions caused noticeable phenotypic differences among the groups (old vs young, fed vs starved, fluorescent marker differences, etc). Blinding during data analysis was not relevant in most cases because automated analysis software was used uniformly to quantify microscopy.

Reporting for specific materials, systems and methods

We require information from authors about some types of materials, experimental systems and methods used in many studies. Here, indicate whether each material, system or method listed is relevant to your study. If you are not sure if a list item applies to your research, read the appropriate section before selecting a response.

Materials & experimental systems

n/a	Involved in the study
☒	☐ Antibodies
☒	☐ Eukaryotic cell lines
☒	☐ Palaeontology and archaeology
☐	☒ Animals and other organisms
☒	☐ Clinical data
☒	☐ Dual use research of concern
☒	☐ Plants

Methods

n/a	Involved in the study
☒	☐ ChIP-seq
☒	☐ Flow cytometry
☒	☐ MRI-based neuroimaging

Animals and other research organisms

Policy information about studies involving animals; ARRIVE guidelines recommended for reporting animal research, and Sex and Gender in Research

Laboratory animals	For all experiments using Caenorhabditis elegans, hermaphroditic animals were used. For experiments using Drosophila 3rd instar larvae, animals were randomly selected irrespective of sex. Ages of adult animals are specifically defined in figures and figure legends for age-specific studies and lifespan analyses. For C. elegans aging experiments 1-2 days of adulthood were used for "early-life" age-points, 5-7 days of adulthood were used for "mid-life" age-points, and 9-10 days of adulthood were used for "late-life" age-points. For experiments using larval C. elegans, L1 larvae were used for all experiments. Supplementary Table 1 provides a list of all C. elegans strains used in this study. The invertebrate fly Drosophila melanogaster was also used to assess conservation of tubular-lysosome induction upon starvation. 3rd instar larvae were used for all Drosophila experiments.
Wild animals	No wild animals were used in this study.
Reporting on sex	For all experiments using Caenorhabditis elegans, hermaphroditic animals were used. For experiments using Drosophila 3rd instar larvae, animals were randomly selected irrespective of sex.
Field-collected samples	No field-collected samples were used in this study.
Ethics oversight	C. elegans and Drosophila research does not require IACUC approval.

Note that full information on the approval of the study protocol must also be provided in the manuscript.
